# An immediate-return reinforcement learning for the atypical Markov decision processes

**DOI:** 10.3389/fnbot.2022.1012427

**Published:** 2022-12-13

**Authors:** Zebang Pan, Guilin Wen, Zhao Tan, Shan Yin, Xiaoyan Hu

**Affiliations:** ^1^State Key Laboratory of Advanced Design and Manufacturing for Vehicle Body, Hunan University, Changsha, Hunan, China; ^2^School of Mechanical Engineering, Yanshan University, Qinhuangdao, Hebei, China

**Keywords:** reinforcement learning, atypical Markov decision process, flight trajectory control, uncertain environments, continuous action space

## Abstract

The atypical Markov decision processes (MDPs) are decision-making for maximizing the immediate returns in only one state transition. Many complex dynamic problems can be regarded as the atypical MDPs, e.g., football trajectory control, approximations of the compound Poincaré maps, and parameter identification. However, existing deep reinforcement learning (RL) algorithms are designed to maximize long-term returns, causing a waste of computing resources when applied in the atypical MDPs. These existing algorithms are also limited by the estimation error of the value function, leading to a poor policy. To solve such limitations, this paper proposes an immediate-return algorithm for the atypical MDPs with continuous action space by designing an unbiased and low variance target Q-value and a simplified network framework. Then, two examples of atypical MDPs considering the uncertainty are presented to illustrate the performance of the proposed algorithm, i.e., passing the football to a moving player and chipping the football over the human wall. Compared with the existing deep RL algorithms, such as deep deterministic policy gradient and proximal policy optimization, the proposed algorithm shows significant advantages in learning efficiency, the effective rate of control, and computing resource usage.

## Introduction

Inspired by the learning pattern of humans, i.e., learning by interacting with the external environment, the concepts of reinforcement learning (RL) were first proposed by Minsky (1954). Subsequently, Bellman (1957) presented a method to define an RL problem using Markov decision processes (MDPs). As a result, an RL problem can be described clearly in terms of states, actions, and rewards. In recent years, with an in-depth combination of deep learning, traditional RL has evolved into deep RL. Generally speaking, deep RL algorithms can be subdivided into value-based algorithms and policy gradient algorithms. Deep Q Network (DQN) was the first exploration for value-based algorithms (Mnih et al., 2015). It solved the dimension explosion problem. Subsequently, various improved DQN algorithms were developed, such as Double DQN (Van Hasselt et al., 2016), Dueling DQN (Wang et al., 2016), etc. However, value-based algorithms could only be applied in discrete rather than continuous action space. In contrast, policy gradient algorithms could solve the RL problem with continuous action space, as an independent actor was constructed to output actions. Note that policy gradient algorithms were generally divided into stochastic policy algorithms and deterministic policy algorithms. The stochastic policy algorithms could output the probability distribution of the actions, such as the asynchronous advantage actor-critic (A3C) (Mnih et al., 2016) and proximal policy optimization (PPO) (Schulman et al., 2017). The deterministic policy algorithms could output the deterministic actions, such as deep deterministic policy gradient (DDPG) (Lillicrap et al., 2015). Due to the advantages of model-free, great self-learning ability, etc., the RL has shown excellent performance in the application of complex control processes. For example, the RL methods were applied to robot manipulators to solve trajectory planning under complex environments (Chen et al., 2022). Tutsoy and Brown studied the RL in problems with Chaotic dynamics and proved that a reasonable discount factor could avoid singular learning problems (Tutsoy and Brown, 2016). Pan et al. (2023) designed a controller for a three-link biped robot using the twin delayed deep deterministic policy gradient algorithm (TD3). Sharbafi et al. designed controllers based on the RL for their football robots and won third place in the 2011 world games (Sharbafi et al., 2011). Massi et al. (2022) increase the learning speed of a navigating robot to improve its performance using the RL method. Even in the financial sector, the RL could be used to learn investment trading policy (Lee et al., 2021). Such trading systems based on RL improved trading performance effectively.

Indeed, the above application scenarios belong to the standard MDPs, containing a series of state transitions. However, the atypical MDP case, which involves only one state transition in continuous action space, can also arise in the engineering field, such as the stamping process (Wang and Budiansky, 1978), directional blasting (Zhu et al., 2008), football trajectory control (Myers and Mitchell, 2013), approximations of the compound Poincaré maps (Li et al., 2020), etc. In such atypical MDPs, the control goal is to maximize the immediate returns rather than the long-term returns. Therefore, compared to the standard MDPs, the atypical MDPs can exhibit many new characteristics. Furthermore, to the best knowledge of the authors, all existing RL algorithms are designed for the standard MDPs to maximize long-term returns. Applying the existing RL algorithms to the atypical MDPs shall lead to the following problems. On the one hand, the existing RL algorithms are also limited by their open problem, i.e., the estimation error of the value function. For example, the sampling errors caused by incomplete samplings will lead to bias for the estimated state-value function (e.g., A3C and PPO) (Mnih et al., 2016; Schulman et al., 2017). For the estimated action-value function, DQN and DDPG can cause the overestimation due to the max operation in off-policy temporal-difference (TD) learning (Mnih et al., 2015; Van Hasselt et al., 2016). In comparison, the TD3 and double DQN may lead to underestimation as the minimum output of two independent target critic networks is selected to update the action-value function (Lillicrap et al., 2015; Fujimoto et al., 2018). Furthermore, the uncertain environment may bring a high variance for the estimated value functions as the uncertainties can lead to entirely different rewards for the same state-action pair. Since the policy gradient formulation is directly related to the value function, the estimation error of the value function can lead to a poor policy and limit the performance of the existing RL algorithms. On the other hand, as the atypical MDPs focus only on immediate returns, the common designs for calculating long-term returns are redundant in the existing RL algorithms. It may result in a waste of computing resources. Moreover, existing algorithms do not notice the difference between estimating the state-value function and the action-value function in atypical MDPs. Such difference determines which approach is more suitable for dealing with atypical MDPs. Thus, regarding the above problems of the existing RL algorithms, this paper aims to propose an immediate-return RL algorithm for atypical MDPs with continuous action space.

On this basis, this paper further takes the football trajectory control as the illustration example to present the superior performance of the proposed algorithm. Indeed, the football trajectory control shall be an ideal test case for the proposed algorithm. The reasons are as follows. As the whole process contains only one state transition from take-off to end and its action, i.e., the football's initial velocity, is continuous, football flight is an atypical MDP case with continuous action space. Meanwhile, the aerodynamic model of football is strongly non-linear and has no analytical solutions (Myers and Mitchell, 2013; Javorova and Ivanov, 2018), which involves many complex physical laws (Horowitz and Williamson, 2010; Norman and McKeon, 2011; Javorova and Ivanov, 2018; Kiratidis and Leinweber, 2018). It is difficult for the traditional control method to control football flight (Hou and Wang, 2013; Hou et al., 2016). Thus, as a challenging task, football trajectory control is an ideal example to test the proposed algorithm. In addition, related researches also have practical application value. The accuracy of the shot is a key of the football robot. Designing a high-performance controller based on the proposed algorithm can promote the development of high-level football robots in the Robot world cup (Sharbafi et al., 2011).

The main contents and contributions of this paper are summarized as the following aspects. Firstly, the characteristics of the atypical MDPs are analyzed systematically based on the RL theory. The disadvantage of estimating the state-value function in the atypical MDPs is explained qualitatively, i.e., the large samples requirement and the unavoidable sampling error. These studies indicate the way to the development of RL algorithms in the atypical MDPs. That is, the deterministic policy has natural advantages in dealing with the atypical MDPs in continuous action space. Secondly, based on the deterministic policy and estimated action-value function, an immediate-return RL algorithm is proposed for the atypical MDPs. In the proposed algorithm, the average reward method is developed to construct an unbiased and low variance target Q-value. Compared with existing RL algorithms, e.g., DDPG and PPO, the proposed algorithm reduces the estimation error significantly. More details are introduced in following Section Immediate-return RL algorithm for the atypical MDPs. Meanwhile, a simplified network framework is also designed for the proposed algorithm. Thus, the proposed decreases both the space complexity and time complexity. The comparison tests also demonstrate that the computing resource consumed by the proposed algorithm is lower than the DDPG and PPO. Thirdly, two challenging scenarios of the football trajectory control, i.e., passing the football to a moving player, and chipping the football over the human wall (chip kick), are presented to test the feasibility of the proposed algorithm. These scenarios can be used as the benchmark to test the algorithms designed for the atypical MDPs. Meanwhile, the controllers based on the proposed algorithm in this paper can improve the football robot's shot accuracy in competitions, such as the Robot world cup (Sharbafi et al., 2011). In the above scenarios, existing RL algorithms (i.e., DDPG, PPO) are also tested as references. Numerical results demonstrate that the immediate-return RL algorithm has higher learning efficiency, a higher effective rate of control, and lower computing resource usage than the reference RL algorithms.

The rest of the present work is organized as follows. In Section The atypical MDPs, the analysis of the atypical MDPs is introduced. Then, the immediate-return RL algorithm for the atypical MDPs is proposed in Section Immediate-return RL algorithm for the atypical MDPs. In Section Illustration examples: Football trajectory control for different scenarios, two illustration examples in MDPs, i.e., passing the football to a moving player and chipping the football over the human wall, are designed. In Section Comparison and discussion, the feasibility and high performance of the RL controllers are demonstrated by simulation tests. And the advantages of the immediate-return RL algorithm are discussed by comparison with the existing RL algorithms. Lastly, the conclusion of this paper is drawn in Section Conclusion.

## The atypical MDPs

### Atypical MDPs: Definition and characteristic analyses

For the standard MDP, it can be described by the states *s*_*t*_, actions *a*_*t*_, and rewards *r*_*t*_ (immediate return). Thus, the trajectory of a standard MDP case contains a series of contiguous state transitions, which can be expressed as follows.

(1) $$\begin{array}{l} \left(s_{0},\;a_{0},\;r_{0}\right)\to \ldots \to \left(s_{t},\;a_{t},\;r_{t}\right)\to \left(s_{t+1},\;a_{t+1},\;r_{t+1}\right)\to \\ \;\ldots \to s_{ter} \end{array}$$

where *s*_*ter*_ is the termination state. Based on RL theory, the state-value function *V*_π_ and action-value function *Q*_π_ in standard MDPs is defined as follows (Watkins, 1989; Sutton and Barto, 2018).

(2) $$\begin{array}{l} V_{\pi }\;(s_{t})=\sum_{a_{t}}\pi \;(a_{t}|s_{t})\sum_{s_{t+1},r_{t}}p\;(s_{t+1},r_{t}|s_{t},a_{t})\\ \;\left[r_{t}+\gamma V_{\pi }\;(s_{t+1})\right] \end{array}$$

(3) $$\begin{array}{l} Q_{\pi }\;(s_{t},\;a_{t})=\sum_{s_{t+1},r_{t}}p\;(s_{t+1},r_{t}|s_{t},a_{t})\\ {}[r_{t}+\gamma \sum_{a_{t+1}}\pi \;(a_{t+1}|s_{t+1})Q_{\pi }\;(s_{t+1},a_{t+1})] \end{array}$$

where *p* is the state transition probability and γ is the reward discount factor (Sutton and Barto, 2018). As shown in Equations (2), (3), both *V*_π_(*s*_*t*_) and *Q*_π_(*s*_*t*_, *a*_*t*_) are closely related to the value of its possible successor states (or state-action pairs) (Sutton and Barto, 2018). Then, the control goal in a standard MDP case is achieving the optimal expected long-term returns. The optimal policy π^*^ can be written as follows (Sutton and Barto, 2018).

(4) $$\begin{array}{l} {\;\pi }^{*\;}\left(s_{t}\right)={\text{argmax}}_{a_{t}\epsilon A}Q_{{\;\pi }^{*}}\;\left(s_{t},\;a_{t}\right) \end{array}$$

In contrast, the atypical MDP case considered in this paper involves continuous action space and has only one state transition from the initial state *s*_*t*_ (*t* = 0) to the termination state *s*_*ter*_. That is, for any state *s*_*t*_, its next state *s*_*t*+1_ is identical to the termination state *s*_*ter*_ after a state transition, i.e., *s*_*t*+1_ ≡ *s*_*ter*_. Its trajectory can be expressed as follows.

(5) $$\begin{array}{l} \left(s_{t},\;a_{t},\;r_{t}\right)\to s_{ter} \end{array}$$

As defined in Equation (5), due to *s*_*t*+1_ ≡ *s*_*ter*_, the whole process of an atypical MDP case only contains one reward *r*_*t*_ (immediate return). Thus, in the atypical MDPs, only the immediate return rather than the long-term return should be considered. Note that the atypical MDP case involving continuous action space is common in engineering field, e.g., stamping process, directional blasting, football trajectory control, approximations of the compound Poincaré maps, etc.

Then, the characteristics of atypical MDPs will be analyzed by comparing the differences between the standard value functions in Equations (2), (3) and the value functions of the atypical MDPs. As defined by Sutton et al., both the state-value and the Q-value at the termination state *s*_*ter*_ are identical to zero (Sutton and Barto, 2018), i.e., *V*_π_(*s*_*ter*_) ≡ 0 and *Q*_π_(*s*_*ter*_) ≡ 0. Since *s*_*t*+1_ ≡ *s*_*ter*_ in the atypical MDPs, the state-value function $V_{\pi }^{A}$ in the atypical MDPs can be written as follows.

(6) $$\begin{array}{l} V_{\pi }^{A}\left(s_{t}\right)=\sum_{a_{t}}\pi \left(a_{t}|s_{t}\right)\sum_{s_{t+1},r_{t}}p\left(s_{t+1},r_{t}|s_{t},a_{t}\right)r_{t}\\ \;=\sum_{a_{t}}\pi \left(a_{t}|s_{t}\right)R\left(s_{t},a_{t}\right) \end{array}$$

In atypical MDPs, $V_{\pi }^{A}\left(s_{t}\right)$ denotes the expected immediate return of the state *s*_*t*_ under the policy π. *R*(*s*_*t*_, *a*_*t*_) is the expected immediate return for the state-action pairs. Compared to the *V*_π_ in standard MDPs [see Equation (2)], although computing the value of $V_{\pi }^{A}$ in the atypical MDPs is independent of its successor state-value $V_{\pi }\left(s_{t+1}\right),\;V_{\pi }^{A}$ is still a function of the policy π in the atypical MDPs. Due to the operation $\underset{a_{t}}{\sum }\pi \left(a_{t}|s_{t}\right)$ in Equation (6), estimating $V_{\pi }^{A}\left(s_{t}\right)$ should traverse the whole action space *A* under the current policy π. It means that approximating the $V_{\pi }^{A}\left(s_{t}\right)$ requires large amounts of samplings when the policy π is stochastic. A finite number of samplings may ignore the huge un-sampled action space and cause an enormous sampling error. Here, suppose that the whole action space *A* consists of the sampled action space *A*^*s*^ and the un-sampled action space *A*^*un*^, i.e., *A* = *A*^*s*^ + *A*^*un*^. Based on Equation (6), there must be a sampling error *err*(*s*_*t*_) between the estimated state-value function $V_{\pi }^{E}$ and true state-value function $V_{\pi }^{A}$, i.e.,

(7) $$\begin{array}{l} V_{\pi }^{A}\left(s_{t}\right)=V_{\pi }^{E}\left(s_{t}\right)+err\left(s_{t}\right) \end{array}$$

where, $V_{\pi }^{E}\left(s_{t}\right)$ and *err*(*s*_*t*_) can be expressed as follows:

(8) $$\begin{array}{l} V_{\pi }^{E}\left(s_{t}\right)=\sum_{a_{t}\in A^{s}}\pi \left(a_{t}|s_{t}\right)R\left(s_{t},a_{t}\right) \end{array}$$

(9) $$\begin{array}{l} err\left(s_{t}\right)=\sum_{a_{t}\in A^{un}}\pi \left(a_{t}|s_{t}\right)R\left(s_{t},a_{t}\right) \end{array}$$

Actually, in standard MDPs, such sampling errors also exist in the estimation of the *V*_π_ and *Q*_π_ since they are also the functions of the policy π. This sampling error introduces the bias for the estimated $V_{\pi }^{E}\left(s_{t}\right)$ and further negatively affect the stochastic policy update. Based on the actor-critic method with baseline (Sutton and Barto, 2018; Levine et al., 2020), the estimated stochastic policy gradient ${\hat{g}}^{E}$ can be written as follows when the biased estimate $V_{\pi }^{E}$ is used (Sutton et al., 1999; Schulman, 2016).

(10) $$\begin{array}{l} {\hat{g}}^{E}=E[\overset{\infty }{\underset{t=0}{\sum }}\left(r_{t}+\gamma V_{\pi }^{E}\left(s_{t+1}\right)-V_{\pi }^{E}\left(s_{t}\right)\right)\nabla_{\omega }\log\pi_{\omega }\left(a_{t}|\;s_{t}\right)]\\ =E[\overset{\infty }{\underset{t=0}{\sum }}(r_{t}+\gamma \left(V_{\pi }^{A}\left(s_{t+1}\right)-err\left(s_{t+1}\right)\right)-(V_{\pi }^{A}\left(s_{t}\right)\\ -err\left(s_{t}\right)))\nabla_{\omega }\log\pi_{\omega }\left(a_{t}|\;s_{t}\right)]\\ =E[\overset{\infty }{\underset{t=0}{\sum }}(\left(r_{t}+{\gamma V}_{\pi }^{A}\left(s_{t+1}\right)-V_{\pi }^{A}\left(s_{t}\right)\right)-(\gamma err\left(s_{t+1}\right)\\ -err\left(s_{t}\right)))\nabla_{\omega }\log\pi_{\omega }\left(a_{t}|\;s_{t}\right)]\\ =\hat{g}+E\left[\overset{\infty }{\underset{t=0}{\sum }}\left(err\left(s_{t}\right)-\gamma err\left(s_{t+1}\right)\right)\nabla_{\omega }\log\pi_{\omega }\left(a_{t}|\;s_{t}\right)\right] \end{array}$$

where $\hat{g}$ is the true stochastic policy gradient. The biased estimate $V_{\pi }^{E}$ causes an ineradicable policy gradient error $\hat{g}$_*err*_ between the estimated $\hat{g}$^*E*^ and true $\hat{g}$, i.e.,

(11) $$\begin{array}{l} {\hat{g}}_{err}=E\left[\overset{\infty }{\underset{t=0}{\sum }}\left(err\left(s_{t}\right)-\gamma err\left(s_{t+1}\right)\right)\nabla_{\omega }\log\pi_{\omega }\left(a_{t}|\;s_{t}\right)\right] \end{array}$$

This error $\hat{g}$_*err*_ may cause negative effects on policy updates.

Under the theory of RL, the action-value function *Q*^*A*^ in the atypical MDPs can be written as follows.

(12) $$\begin{array}{l} Q^{A}\left(s_{t},\;a_{t}\right)=\sum_{s_{t+1},r_{t}}P\left(s_{t+1},r_{t}|s_{t},a_{t}\right)r_{t}=R\left(s_{t},a_{t}\right) \end{array}$$

In the atypical MDPs, $Q^{A}\left(s_{t},\;a_{t}\right)$ denotes the expected immediate return of the state-action pairs (*s*_*t*_, *a*_*t*_). And the action-value function *Q*^*A*^ is also unrelated to the value of its successor state-action pairs as same as the $V_{\pi }^{A}$ in Equation (6). However, it should be particularly stressed that the action-value function *Q*^*A*^ in the atypical MDPs is a function independent of policy π, which is different from the *V*_π_ in Equation (2), *Q*_π_ in Equation (3), and $V_{\pi }^{A}$ in Equation (6). Thus, it brings a set of new characteristics for the *Q*^*A*^ as follows. Firstly, the value of the $Q^{A}\left(s_{t},\;a_{t}\right)$ will not be changed in policy updating. However, with the policy π updating, the state-value function $V_{\pi }^{A}$ in the atypical MDPs will be changed accordingly. That is, compared to approximating the *Q*^*A*^, approximating the $V_{\pi }^{A}$ requires more samples and more training steps. Meanwhile, since there is no $\underset{a_{t}}{\sum }\pi \left(a_{t}|s_{t}\right)$ operation in Equation (12), it is unnecessary for estimating the action-value function *Q*^*A*^ to traverse the whole action space. It also indicates that much more samples are required to estimate $V_{\pi }^{A}\left(s_{t}\right)$ than to estimate $Q^{A}\left(s_{t},\;a_{t}\right)$ in an atypical MDP case. This also indicates that estimating the $V_{\pi }^{A}\left(s_{t}\right)$ in an atypical MDPs requires more samples than the Q-function. Thus, estimating the state-value function can lead to the low learning efficiency of the RL algorithms. Secondly, the bias caused by sampling error will not exist in the estimated action-value function *Q*^*E*^ as Equation (12) does not contain operation $\underset{a_{t}}{\sum }\pi \left(a_{t}|s_{t}\right)$. In contrast, such bias is inevitable for the estimated state-value function $V_{\pi }^{E}$, as discussed in Equation (7). Based on the above analysis, estimating the $Q^{A}\left(s_{t},\;a_{t}\right)$ is easier than estimating the $V_{\pi }^{A}\left(s_{t}\right)$ in the atypical MDPs. Generally speaking, the stochastic policy algorithms rely on the estimated state-value function $V_{\pi }^{E}$, and the deterministic policy algorithms rely on the estimated action-value function *Q*^*E*^. Thus, when dealing with the atypical MPD case, deterministic policy algorithms can show more natural advantages than the stochastic policy algorithms.

In addition, the new characteristic of the atypical MDP case is also shown in its policy π^*^. Based on the definition of the action-value function *Q*^*A*^ in Equation (12), the optimal policy π^*^ under the atypical MDPs can be expressed as follows.

(13) $$\begin{array}{l} {\;\pi }^{*}\left(s_{t}\right)=\underset{a_{t}\in A}{\text{argmax}}\sum_{s_{t+1},r_{t}}P\left(s_{t+1},r_{t}|s_{t},a_{t}\right)r_{t}\\ \;=\underset{a_{t}\in A}{\text{argmax}}R\left(s_{t},\;a_{t}\right) \end{array}$$

That is, in the atypical MDPs, the goal of the optimal policy π^*^ is achieving the maximal expected reward rather than the maximal expected long-term returns. And the long-term returns can be ignored for policy update in the atypical MDPs.

### Limitations of existing RL algorithms in the atypical MDPs

When dealing with the atypical MDP cases in continuous action space, the existing RL algorithms are limited by their open problems as well as by the special problems caused by the characteristic of the atypical MDP case. Note that the value-based algorithms will not be discussed here as they are only applicable to discrete action space.

The estimation error of the estimated value function, i.e., bias and variance, is an open problem that limits the performance of RL algorithms. The bias may be introduced to the estimated value function based on TD learning due to the off-policy TD learning's max operation, chosen imperfect policy, and uncertainties (Sutton and Barto, 2018). TD learning method is an important estimation method for the value function and is widely used in existing RL algorithms, e.g., PPO, DDPG, etc. Especially for deterministic policy algorithms, e.g., DDPG, TD learning's max operation may lead to an overestimated Q-value (Van Hasselt et al., 2016), bringing negative effects to the policy update. Although the TD3 (Fujimoto et al., 2018) improves the overestimation, TD3 may lead to the underestimated Q-value and increase the complexity of the algorithm significantly. Additionally, as analyzed in Equation (7), sampling error caused by incomplete samplings can further increase the bias for the stochastic policy algorithms that rely on the estimated state-value function $V_{\pi }^{E}$, e.g., A3C, PPO, etc. Note that some complex scenario involving uncertain environments may generate completely different response results for the same state-action pair. Such complex and uncertain responses can bring a high variance for the estimated value functions, leading low reliability of controller. However, existing RL algorithms do not solve this problem very well.

As analyzed in Equation (13), a characteristic of the atypical MDPs is that they focus only on the maximum immediate return. And there is no focus on the long-term return. However, there are many designs for estimating long-term returns in existing RL algorithms. For example, based on Equations (2), (3), many existing RL algorithms, such as PPO, DDPG, etc., have an operation to calculate the successor state-value (or Q-value). When dealing with an atypical MDP case, such an operation is redundant and increases the time complexity of the algorithms, e.g., PPO. Especially for deterministic policy algorithms, e.g., DDPG and TD3, they contain a set of complex target networks to calculate the successor Q-value. It shall increase a great of both time complexity and space complexity. Such limitations can increase computing resource usage, which is not conducive to applying RL algorithms to complex problems.

## Immediate-return RL algorithm for the atypical MDPs

### The immediate-return RL algorithm

**Figure 1:**
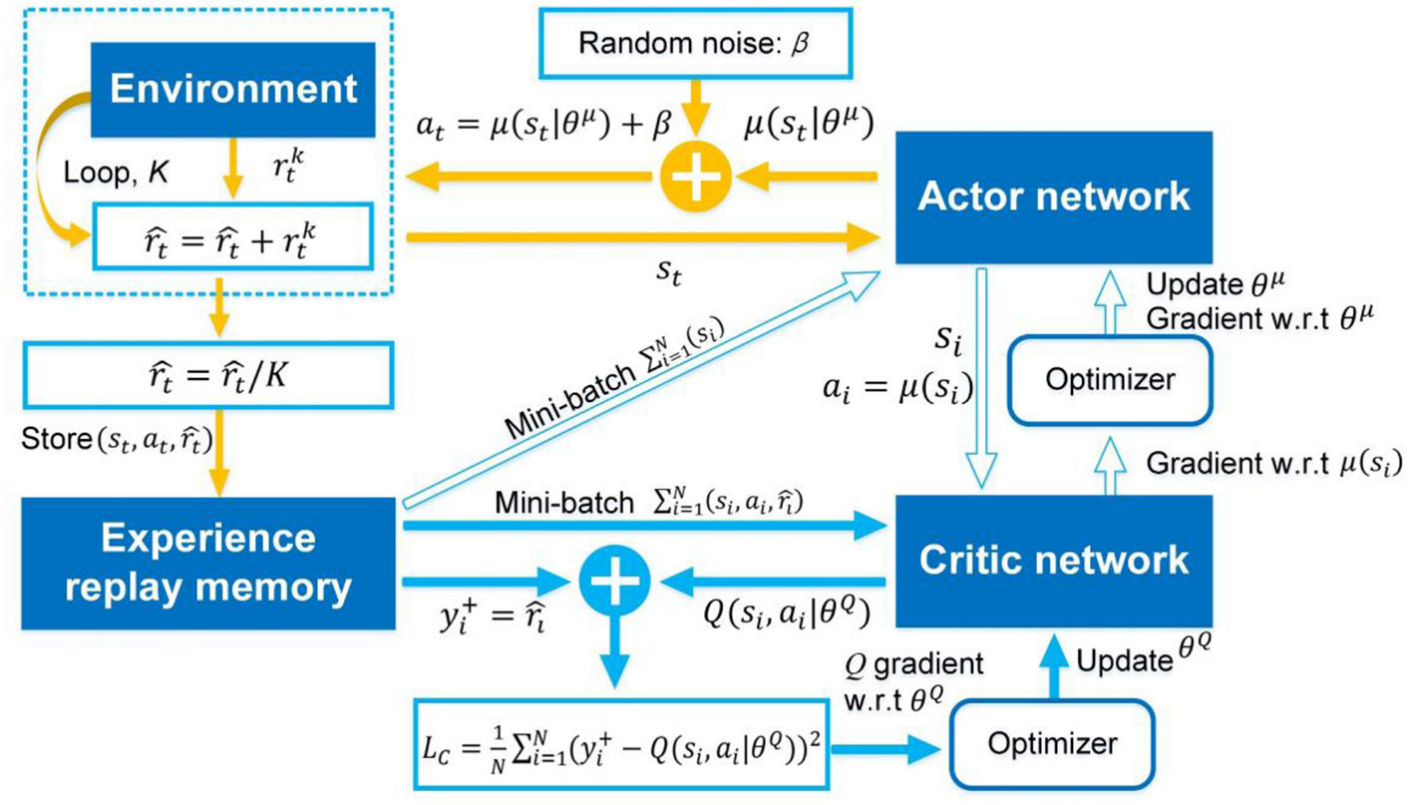
The framework of the immediate-return RL algorithm. Yellow solid arrows: the actor network interacts with the environment. Blue solid arrow: update for critic network. Blue hollow arrow: update for actor network.

As analyzed in Section The atypical MDPs, deterministic policy shows more advantages than stochastic policy in atypical MDPs. Thus, the immediate-return RL algorithm is proposed based on the deterministic policy method and actor-critic framework for the problems in atypical MDPs. The new equations involved in this algorithm are highlighted in “⇐”. As shown in Figure 1, two networks, i.e., actor network with weights θ^μ^ and critic network with weights θ^*Q*^, are designed to construct the actor-critic framework. Here the actor network plays a role as the policy. It can output deterministic action $a_{t}=\mu \left(s_{t}|\theta^{\mu }\right)$ based on the inputted state *s*_*t*_. The critic network is used as the estimated action-value function. It can evaluate the performance of the actor network by outputted the estimated Q-value $Q\left(s_{t},\;a_{t}|\theta^{Q}\right)$ according to the inputted state-action pair (*s*_*t*_, *a*_*t*_). Compared to other deterministic policy algorithms (e.g., DDPG), the proposed algorithm's network framework has been simplified significantly due to no target networks. It means less computing resource usage.

As analyzed in Equation (12), the true action-value function *Q*^*A*^ in atypical MDPs is equal to the expected reward *R*(*s*_*t*_, *a*_*t*_). As shown in Equation (13), the immediate reward *r*_*t*_ (i.e., immediate return) is the unbiased estimation for the expected reward *R*(*s*_*t*_, *a*_*t*_).

(14) $$\begin{array}{l} E_{r_{t},s_{t+1}}\left[r_{t}\right]=\sum_{s_{t+1},r_{t}}P\left(s_{t+1},r_{t}|s_{t},a_{t}\right)r_{t}=R\left(s_{t},a_{t}\right)⇐ \end{array}$$

When the environment is deterministic, the generated next state *s*_*t*+1_ and immediate reward *r*_*t*_ are also deterministic under the specified state-action pair (*s*_*t*_, *a*_*t*_). Under this condition, the immediate reward *r*_*t*_ is equal to its expectation, i.e., *r*_*t*_ = *R*(*s*_*t*_, *a*_*t*_). Thus, *r*_*t*_ is the ideal target Q-value $y_{t}^{+}$ of the estimated action-value function in an atypical MDP with a deterministic environment. However, the uncertain environments (e.g., dynamic systems with uncertainties) may generate different immediate rewards *r*_*t*_ even given the same state-action pair (*s*_*t*_, *a*_*t*_). A randomly generated reward value *r*_*t*_ cannot represent the expected reward *R*(*s*_*t*_, *a*_*t*_) under the specified state-action pair (*s*_*t*_, *a*_*t*_). Due to the complexity of the uncertainties, the probability distribution of these generated reward values is also unknown. Therefore, using *r*_*t*_ as the critic network's target *Q*-value will result in a high estimation variance when considering uncertainties. The high variance may lead to instability in the learning process, making the policy less reliable (Fujimoto et al., 2018; Sutton and Barto, 2018). Based on the law of large numbers, the average reward $\hat{r_{t}}$ is proposed as the target Q-value to solve the problem of high variance caused by uncertain environments. $\hat{r_{t}}$ can be expressed as follows.

(15) $$\begin{array}{l} \hat{r_{t}}=\frac{1}{K}\sum_{k=1}^{K}r_{t}^{k}⇐ \end{array}$$

That is, a specified state-action pair (*s*_*t*_, *a*_*t*_) will be performed multiple times *K* in the uncertain environment. And a set including multiple immediate rewards $\left\{r_{t}^{k}\right\}$ will be obtained. This immediate reward set $\left\{r_{t}^{k}\right\}$ can reflect the probabilistic characteristics of the uncertain environment's responses under the state-action pair (*s*_*t*_, *a*_*t*_). Then, the average reward $\hat{r_{t}}$ is constructed by averaging the immediate reward set $\left\{r_{t}^{k}\right\}$. According to the law of large numbers, the average reward $\hat{r_{t}}$ is closer to the expected reward *R*(*s*_*t*_, *a*_*t*_) than one randomly generated reward *r*_*t*_. Thus, there will be minor variance, when the average reward $\hat{r_{t}}$ is used to estimate the expected reward *R*(*s*_*t*_, *a*_*t*_). Note that the average reward $\hat{r_{t}}$ is still the unbiased estimation for the expected reward *R*(*s*_*t*_, *a*_*t*_) due to the average operation. In practical application, the repetition number *K* is suggested to be 3 based on experience. In the football trajectory control problems considered in this paper, setting the repetition number *K* to 3 can significantly improve the training results compared to setting the number of repetitions to 1. When *K* continues to increase, the algorithm's performance cannot be significantly improved. Based on the above analysis, these improvements provide an unbiased and low variance target *Q*-value for the critic network. It can make the proposed algorithm more reliable in uncertain environments. The problem of the estimation error in the existing RL algorithms, e.g., overestimation in DDPG, can also be overcome. The numerical tests in Section Controller's performance will prove this. Then, the new target Q-value $y_{t}^{+}$ of the immediate-return RL is expressed as

(16) $$\begin{array}{l} y_{t}^{+}=\hat{r_{t}}⇐ \end{array}$$

It should be noted that the average reward $\hat{r_{t}}$ applies only to the atypical MDP case, as the successor state *s*_*t*+1_ relying on the current state-action pair (*s*_*t*_, *a*_*t*_) does not exist.

The off-policy method (Levine et al., 2020) is also adopted in the proposed algorithm. All training samples generated from the trial and error should be stored in the experience memory. It should be stressed that the atypical MDP case does not focus on the successor state *s*_*t*+1_, and its trajectory contains only one state transition. Hence, only the initial samples of each trajectory, i.e., ($s_{t},\;a_{t},\;\hat{r_{t}}$), *t* ≡ 0, should be stored. Sampling *N* training samples $\sum_{i\;=\;1}^{N}\left(s_{i},\;a_{i},\hat{r_{i}}\right)$, the loss function for updating critic network is expressed as follows.

(17) $$\begin{array}{l} L_{C}=\frac{1}{N}\sum_{i\;=\;1}^{N}(y_{i}^{+}-Q{\left(s_{i},a_{i}|\theta^{Q})\right)}^{2}\;|y_{i}^{+}=\hat{r_{i}} \end{array}$$

where *N* is the size of the min-batch. Symbol *i* is the label number of the sample. By minimizing the loss function, the critic network weights θ^*Q*^ can be updated. Meanwhile, as analyzed in Equation (13), the policy should be updated in the direction of maximizing the expected reward. Thus, the purpose of updating the actor network μ is to maximize the estimated Q-value outputted by the critic network. Referring to Lillicrap et al. (2015), the gradient for updating actor network weights θ^μ^ is expressed as follows.

(18) $$\begin{array}{l} \nabla_{\theta^{\mu }}{|}_{s_{i}}=\frac{1}{N}\sum_{i=1}^{N}\nabla_{a_{i}}Q\left(s_{i},a_{i}=\mu \left(s_{i}\right)|\theta^{Q}\right)\;\nabla_{\theta^{\mu }}\mu \left(s_{i}|\theta^{\mu }\right) \end{array}$$

Furthermore, the delaying policy update (Fujimoto et al., 2018) is also introduced for the immediate-return RL algorithm. It can reduce the frequent policy updates and further result in low variance (Fujimoto et al., 2018). After successful training, the actor network will be the RL controller.

In summary, due to the proposed average reward method, the open problem of the estimation error can be improved significantly in the proposed algorithm. Compared to existing RL algorithms, the proposed algorithm will show high performance. This point will be certified in two football trajectory control scenarios (see Section Comparison and discussion). Besides, based on the characteristics of atypical MDPs, a simplified network framework is designed for the proposed algorithm to reduce computing resource usage. Then, the complete pseudocode of the immediate-return RL algorithm is shown in Algorithm 1.

**Algorithm 1:** The immediate-return RL algorithm.

1: Randomly initialize actor network μ with weights θ^μ^
2: Randomly initialize critic network *Q* with weights θ^*Q*^
3: Initialize the experience replay memory *E*
4: **For** step *t*= 1, T **do**
5: Generate initial state *s*_*t*_ in the environment
6: Output action $a_{t}=\mu \left(s_{t}|\theta^{\mu }\right)+\beta$ based on current policy and random noise
7: Initialize average reward $\hat{r_{t}}=0$
8: **For** *k*= 1, *K* **do**
9: Running the state-action pair (*s*_*t*_, *a*_*t*_) in environment on the *kth* times
10: Observe the reward $r_{t}^{\text{k}}$, and $\hat{r_{\text{t}}}=\hat{r_{\text{t}}}+r_{\text{t}}^{\text{k}}$ ⇐
**End Loop *K***
11: Calculate average reward $\hat{r_{\text{t}}}=\hat{r_{\text{t}}}/\text{K}$ ⇐
12: Store the sample $\left(s_{t},\;a_{t},\;\hat{r_{t}}\right)$ in *E*
13: Extract random a minibatch of *N* samples $\overset{N}{\underset{i=1}{\sum }}\left(s_{i},\;a_{i},\hat{r_{i}}\right)$ from *E*
14: Obtain the target Q-value $y_{i}^{+}=\hat{r_{i}}$ ⇐
15: Construct the loss function *L*_*C*_ of the critic network:
$L_{C}=\frac{1}{N}\overset{N}{\underset{i=1}{\sum }}{\left(y_{i}^{+}-Q\left(s_{i},a_{i}|\theta^{Q}\right)\right)}^{2}$
16: Update the critic network weights θ^*Q*^ by minimizing the loss *L*_*C*_
17: **If** *t* mod *d* **then**
18: Update the actor network weights θ^μ^ using the gradient:
$\nabla_{\theta^{\mu }}{|}_{s_{i}}=\frac{1}{N}\overset{N}{\underset{i=1}{\sum }}\nabla_{a_{i}}Q\left(s_{i},a_{i}=\mu \left(s_{i}\right)|\theta^{Q}\right)\;\nabla_{\theta^{\mu }}\mu \left(s_{i}|\theta^{\mu }\right)$ **End IF**
**End Loop *T***

### Complexity analysis

The computing complexity, i.e., space complexity and time complexity, can reflect the requirement of the algorithm for computing resources. To verify the low computing resource requirement of the immediate-return RL algorithm, the computing complexity of the proposed algorithm will be analyzed in this section. Meanwhile, the representative of the stochastic policy algorithms, i.e., PPO, and the representative of the deterministic policy algorithms, i.e., DDPG, will also be analyzed as references. For the details of DDPG and PPO, please see Lillicrap et al. (2015), Schulman et al. (2017). In the following analysis, the single network's detailed architectures in Section Training process will be used as an example for clarity.

Since the algorithms mentioned above are composed of networks, their space complexity depends on the total parameter of all networks. According to Han et al. (2015), the whole space complexity of a single network is:

(19) $$\begin{array}{l} space˜O\left(\sum_{l\;=\;1}^{L-1}N_{l}N_{l+1}+N_{l+1}\right) \end{array}$$

where *L*=5 is the total layer number of the networks. *N*_*l*_ represents the total node number of the *l* layer. As shown in Table 1, both the proposed algorithm and PPO have two networks (Schulman et al., 2017), and the DDPG contains four networks (Lillicrap et al., 2015). Thus, the space complexity of the proposed algorithm is similar to PPO and is reduced by 50% compared to DDPG.

**Table 1:** The space and time complexity analysis.

		**The proposed algorithm**	**DDPG**	**PPO**
Space complexity	Actor network	199,558	199,558	200,332
	Critic network	200,449	200,449	198,913
	Target networks	\	400,007	\
	Total	400,007	800,014	399,245
Time complexity (FLOPs)	Actor network	397,312	397,312	398,848
	Critic network	399,104	399,104	396,032
	Target networks	\	796,416	\
	Once Sampling	397,312	397,312	398,848
	Once network Update	796,416	1,592,832	(794,880~1,190,912)

The time complexity of the RL algorithms depends on both the network framework and the calculation process (i.e., sampling process and update process). Generally, floating point operations (FLOPs) is used to evaluate the algorithm's time complexity. Referring to He and Sun (2015), the time complexity of a single network is:

(20) $$\begin{array}{l} time˜O\left(\sum_{l\;=\;1}^{L-1}{2N}_{l}N_{l+1}\right) \end{array}$$

Then, the time complexity of one sampling and one network update will be discussed separately (see Table 1). For the three algorithms discussed in this article, only the actor network is working when sampling. Thus, the time complexity of the three discussed algorithms can be regarded as the same in one sampling and is equal to the actor network's time complexity (see Table 1). Note that although the state-action pair (*s*_*t*_, *a*_*t*_) will be performed many times in the environment (Algorithm 1 Line 8 to Line 10), the proposed algorithm's time complexity will not be increased in one sampling, as its actor network only runs once. Regarding the time complexity of network updates, only the network's forward computation is considered according to He and Sun (2015). When the proposed algorithm and DDPG (Lillicrap et al., 2015) update their networks, all their networks will be used once. Here, the proposed algorithm has 2 networks, and DDPG has four (Lillicrap et al., 2015). Thus, the proposed algorithm reduces the time complexity of each network update by 50% than DDPG (see Table 1). In each network update, the actor network and critic network of PPO should estimate π(*s*_*t*_) the *V*(*s*_*t*_), respectively (Schulman et al., 2017). Besides, for the same batch of samples that are trained multiple times, the critic network should be used once to estimate *V*(*s*_*t*+1_) due to the TD learning method (Schulman et al., 2017). That is, the fewer times the same batch of samples are trained, the greater the time complexity of each network update. When a batch of samples is used only once, the proposed algorithm can reduce the time complexity of each network update by 33.1% than the PPO (see Table 1). Thus, based on the above analysis, when the sampling times and the network update times are constants, the time complexity of the proposed algorithm is 40% lower than the DDPG and 0–24.9% lower than the PPO.

It should be stressed that computing resources are limited and precious. Especially for some actual complex tasks involving vision, the usage of computing resources is enormous. Based on the above analysis, the immediate-return RL algorithm has lower computing complexity than the existing RL algorithms, reducing computing resource usage. Such statements will be verified in the following Section Computing resource usage by detailed comparisons.

## Illustration examples: Football trajectory control for different scenarios

The football flight is an atypical MDP case. To test the immediate-return RL algorithm, two highly challenging scenarios involving the flight control of the football, i.e., passing the football to a moving player, and chipping the football over the human wall, will be examined. These scenarios can be used as the benchmark to test the algorithms designed for the atypical MDPs. Meanwhile, regarding research results can be used to develop high-level football robots in the Robot world cup (Sharbafi et al., 2011). The controllers based on the proposed algorithm in this paper can significantly increase the accuracy of the football shot.

Under the above two scenarios, the proposed controllers will be trained to output accurate initial velocities for the football to achieve the specified flight purposes and reduce the time of football flight. In the following sections, the experimental model will be introduced in Section Experimental model: Aerodynamic model of football with parameter uncertainties first. Then, other detailed designs corresponding to the two different scenarios, including the actions designs, states designs and constraints, the termination events definitions, and the reward function designs, will be introduced in Section Scenario 1: passing the football to a moving player and Section Scenario 2: chipping the football over the human wall.

### Experimental model: Aerodynamic model of football with parameter uncertainties

Here, an aerodynamic model of football under windless conditions is directly reproduced here from Myers and Mitchell (2013), Javorova and Ivanov (2018). On this basis, parameter uncertainties are newly introduced into the aerodynamic model of the football. Thus, the football flight process can be regarded as an uncertain environment. This aerodynamic model will be adopted directly as the simulation environment to further generate the training data for the RL controllers.

The external forces acting on the ball include gravity **G**, drag force **F**_*D*_, lift force *F*_*L*_, and drag moment **M**_*D*_. Thus, the aerodynamic model of football can be expressed as follows (Myers and Mitchell, 2013; Javorova and Ivanov, 2018).

(21) $$\begin{array}{l} \tilde{m}\ddot{x}=-K_{D}\dot{x}\sqrt{{\dot{x}}^{2}+{\dot{y}}^{2}+{\dot{z}}^{2}}+K_{L}\left({\dot{x}}^{2}+{\dot{y}}^{2}+{\dot{z}}^{2}\right)\\ \;\left(\omega_{Y}\dot{z}-\omega_{Z}\dot{y}\right) \end{array}$$

(22) $$\begin{array}{l} \tilde{m}\ddot{y}=-K_{D}\dot{y}\sqrt{{\dot{x}}^{2}+{\dot{y}}^{2}+{\dot{z}}^{2}}+K_{L}\left({\dot{x}}^{2}+{\dot{y}}^{2}+{\dot{z}}^{2}\right)\\ \;\left(\omega_{Z}\dot{x}-\omega_{X}\dot{z}\right) \end{array}$$

(23) $$\begin{array}{l} \tilde{m}\ddot{z}=-K_{D}\dot{z}\sqrt{{\dot{x}}^{2}+{\dot{y}}^{2}+{\dot{z}}^{2}}+K_{L}\left({\dot{x}}^{2}+{\dot{y}}^{2}+{\dot{z}}^{2}\right)\\ \;\left(\omega_{X}\dot{y}-\omega_{Y}\dot{x}\right)-\tilde{m}\text{g} \end{array}$$

(24) $$\begin{array}{l} {\dot{\omega }}_{X}=-\eta \omega_{X} \end{array}$$

(25) $$\begin{array}{l} {\dot{\omega }}_{Y}=-\eta \omega_{Y} \end{array}$$

(26) $$\begin{array}{l} {\dot{\omega }}_{Z}=-\eta \omega_{Z} \end{array}$$

where parameters *K*_*D*_ and *K*_*L*_ are specified as follows.

(27) $$\begin{array}{l} K_{D}=0.5{\tilde{C}}_{d}\tilde{\rho }\pi {\tilde{r}}^{2} \end{array}$$

(28) $$\begin{array}{l} K_{L}=0.5C_{L}\tilde{\rho }\pi {\tilde{r}}^{2}\frac{1}{\left|\omega \times \text{v}\right|} \end{array}$$

here, $\tilde{m}$ is the football's mass, **g** is the gravitational acceleration, $\tilde{\rho }\;$is the air density, $\tilde{r}$ is the radius of the football, **v=**($\dot{x}$, $\dot{y}$, $\dot{z}$) is the linear velocity, and ω = (ω_*X*_, ω_*Z*_, ω_*Y*_) is the angular velocity. The attenuation coefficient η is assumed to be 0.05. Furthermore, the dimensionless lift coefficient *C*_*L*_ is adopted from Kiratidis and Leinweber (2018) as follows.

(29) $$\begin{array}{l} C_{L}=\left(1-\partial v^{2}\right){S_{p}}^{\beta } \end{array}$$

here, the parameter ∂ is chosen as 2.5 × 10^−4^, and β is 0.83 (Kiratidis and Leinweber, 2018). The spin parameter is $S_{p}=\frac{\tilde{r}\omega }{v}$, where ω = |ω| and *v* = |**v**|. The dimensionless drag coefficient is expressed as $\tilde{C_{d}}$, which is an important factor for the sudden change of linear velocity of football in flight (Horowitz and Williamson, 2010; Norman and McKeon, 2011). Its fitting function is adopted from Kiratidis and Leinweber (2018) as follows.

(30) $$\begin{array}{l} \tilde{C_{d}}\left(v,sp\right)=\frac{a_{c}-b_{\min}}{1+e^{\frac{v-v_{c}}{v_{s}}}}+b_{\min}+\frac{v-v_{\min}}{1+e^{\frac{-v+v_{\min}}{v_{s}}}}\;\frac{b_{\max}-b_{\min}}{v_{\max}-v_{\min}}\\ \;+b_{r}S_{p} \end{array}$$

where *a*_*c*_, *b*_min_, *b*_max_, *b*_*r*_, *v*_min_, *v*_max_*, v*_*c*_, and *v*_*s*_, are the fitting coefficients of the above function (see Table 2).

**Table 2:** The fitting coefficients of the drag coefficient function (Kiratidis and Leinweber, 2018).

**Balls**	***a_*c*_***	***v_*c*_***	***v_*s*_***	***b_*min*_***	***b_*max*_***	***v_*min*_***	***v_*max*_***	***b_*r*_***
Tango12	0.5452	12.8600	1.3040	0.1657	0.1953	16.2200	35.0000	0.5332
Teamgeist	0.4927	12.5800	1.0710	0.1440	0.1540	23.1700	35.0000	0.5140
Brazuca	0.4740	12.9200	1.0000	0.1657	0.2112	14.6100	35.0000	0.5397

These fitting coefficients are derived from the actual wind tunnel data of famous footballs, including Tango12, Teamgeist and Brazuca.

**Figure 2:**
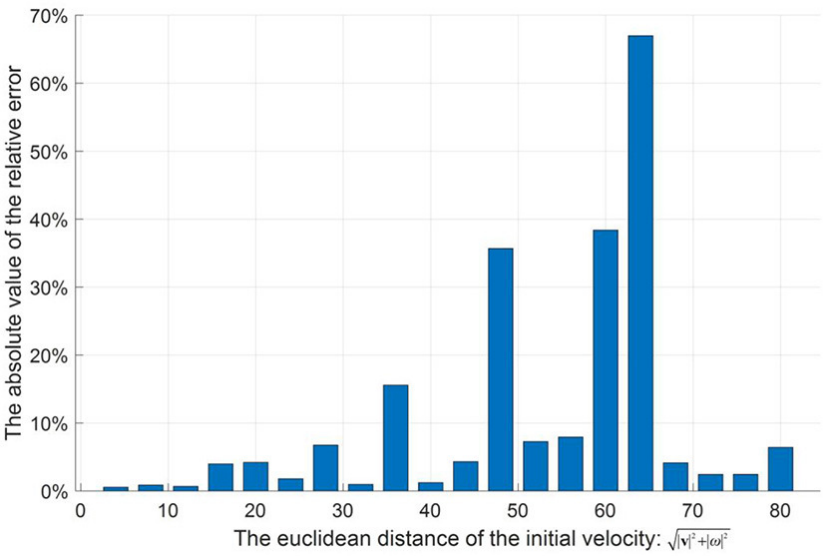
The effect of parameter uncertainty on flight trajectory.

Next, parameter uncertainties, i.e., air density $\tilde{\rho }$, mass $\tilde{m}$, radius $\tilde{r}$, and drag coefficient $\tilde{C_{d}}$, in the aerodynamic model of football will be introduced. Here, $\tilde{m}$, $\tilde{\rho }$, $\tilde{r}$, and $\tilde{C_{d}}$, are internal parameters, and $\tilde{\rho }$ is external parameter. All parameters with uncertainties are random and parametric. The following parameters, i.e., $\tilde{\rho }$, $\tilde{m}$, and $\tilde{r}$, can change in very small intervals according to the international federation of association football (FIFA) standards, and these details are shown in Table 3. In addition, the different fitting coefficients of the drag coefficient functions (Kiratidis and Leinweber, 2018) corresponding to three kinds of footballs, i.e., Tango12, Teamgeist, or Brazuca, are considered in this paper (see Table 2). That is, when giving specified initial conditions and simulating Equations (21)–(26) in the training or testing procedures, values of the $\tilde{\rho }$, $\tilde{m}$, and $\tilde{r}$ will be selected randomly from Table 3, and one set of the fitting coefficients of the drag coefficient function will be selected randomly from the Table 2. Note that slight changes in the above parameters can significantly impact the flight trajectories, although the football has the same initial condition. In order to analyze the impact of the parameter uncertainties, 20 random initial conditions are generated to test. Based on Equations (21)–(26), each initial condition is simulated 100 times and produces 100 flight trajectories. In each initial condition, the average landing position of these 100 flight trajectories is set as the target position. Then, the average relative error of the 100 landing positions relative to the target position can be calculated to assess the impact of the parameter uncertainties. The results of 20 tests are shown in Figure 2. Here, the maximum average relative error is 66.97%. The average value of 20 average relative errors is 10.63%. Thus, parameter uncertainties can have a non-negligible impact on the flight trajectory and pose a significant challenge to the controller design.

**Table 3:** The range of the uncertain parameters.

**Uncertain** **parameters**	**Unit**	**Minimum value**	**Maximum value**
Air density $\tilde{\rho }$	kg/m^3^	1.000	1.205
Mass $\tilde{m}$	kg	0.42	0.45
Radius $\tilde{r}$	m	0.1090	0.1106

### Scenario 1: Passing the football to a moving player

**Figure 3:**
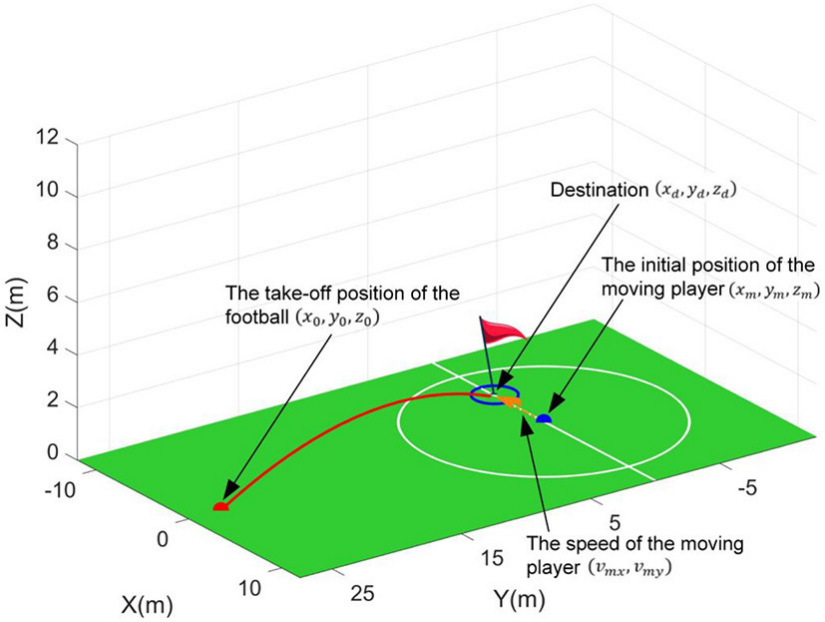
Passing the football to a moving player.

The schematic diagram of the first scenario is shown in Figure 3. And this scenario simulates the dynamic passing situation between the players in reality. That is, the moving player moves when the football flies and stops when the football lands. Here, two control targets, i.e., passing the football to a moving player and reducing the time of the football flight, are set for the RL controller.

The action outputted by the RL controller is the initial velocities of the football, i.e., initial linear velocity and initial angular velocity. This action is designed as follows.

(31) $$\begin{array}{l} A_{0}=\left(v_{x},v_{y},v_{z},\;\omega_{x},\omega_{y},\omega_{z}\right) \end{array}$$

It should be noted that both the linear and angular velocities should be limited according to the practical data of the professional players (Neilson, 2003), i.e., |**v**|∈[0, 34] m/s and |ω| ∈ [0, 62.8] rad/s.

In this scenario, the initial position of the moving player will be set at the coordinate origin for convenience, i.e., (*x*_*m*_, *y*_*m*_, *z*_*m*_) = (0, 0, 0). Thus, the conditions when the football takes off, i.e., state *S*_1_, can be described as follows.

(32) $$\begin{array}{l} S_{1}=\left(x_{0},y_{0},z_{0},v_{mx},v_{my}\right) \end{array}$$

where (*x*_0_, *y*_0_, *z*_0_) is the football's initial take-off position. The (*v*_*mx*_, *v*_*my*_) is the moving speed of the moving player. Then, the constraints for the state *S*_1_ are set as follows. Firstly, according to the player's sprint speed (Djaoui et al., 2017), the maximum speed of moving players is limited to 10 m/s, i.e.,

(33) $$\begin{array}{l} v_{m}=\sqrt{{v_{mx}}^{2}+{v_{my}}^{2}}\leq 10 \end{array}$$

Secondly, considering the size of the sports field, the constraint on the choice of the take-off position is defined as follows.

(34) $$\begin{array}{l} d_{m}=\sqrt{{\left(x_{0}-x_{m}\right)}^{2}+{\left(y_{0}-y_{m}\right)}^{2}+{\left(z_{0}-z_{m}\right)}^{2}}\leq 30 \end{array}$$

Note that the destination (*x*_*d*_, *y*_*d*_, *z*_*d*_) of the football in this scenario is defined as the end position of the moving player, i.e., (*x*_*m*_ + *v*_*mx*_*t*_*f*_, *y*_*m*_ + *v*_*my*_*t*_*f*_, *z*_*m*_). *t*_*f*_ is the football's flight time. That is, the destination is not a constant pre-defined in the state *S*_1_ and unknown for the RL controller. Thus, passing the football to a moving player is a challenging scenario.

To generate reasonable trajectories, some termination events of the simulations should be set according to the constraints required. Any of termination events are triggered, the flight process will be stopped. In this scenario, the ground floor *Z*_*LB*_ = 0 and maximum height *Z*_*LH*_ = 12 are set as the constraints for flight trajectories. Therefore, the termination events for this scenario are defined as *z*_*f*_ = *Z*_*LB*_ or *z*_*f*_ = *Z*_*LH*_. Here, the (*x*_*f*_, *y*_*f*_, *z*_*f*_) denotes the football's final position when the termination event is triggered.

For the purpose of learning an excellent policy to predict proper initial velocities, the RL controller needs to be guided by an appropriate reward function. Here, a monotonic power function (i.e., *y* = 1 − *x*^*b*^) is selected as the basic function to design the reward function. For this basic function, the closer *x* is to 0, the greater the change in the gradient $\frac{dy}{dx}$. Thus, the reward function based on this power function can provide very large positive rewards for a small number of correct samples in some complex scenarios. It may provide more precise guidance for RL algorithms. Note that other function forms may also have similar effects, and the proposed basic functions only offer an effective solution. In this scenario, two sub-reward functions based on this basic function are designed for two independent control targets, i.e., passing the football to a moving player, and reducing the time of football flight, respectively. Then, two sub-reward functions will be combined into one united reward function by reward shaping (Brys et al., 2017) to integrate these two control targets.

For the first control target, i.e., passing the football to a moving player, the relative error δ between the football's final position (*x*_*f*_, *y*_*f*_, *z*_*f*_) and the destination (*x*_*d*_, *y*_*d*_, *z*_*d*_) is a reasonable parameter to evaluate the flight results. It can be expressed as follows,

(35) $$\begin{array}{l} \delta =\Delta d/d_{d} \end{array}$$

where, Δ*d* is the absolute error between the football's final position and the destination, i.e.,


(36) $$\begin{array}{l} \Delta d=\sqrt{{\left(x_{d}{-x}_{f}\right)}^{2}+{\left(y_{d}-y_{f}\right)}^{2}+{\left(z_{d}-z_{f}\right)}^{2}} \end{array}$$

*d*_*d*_ indicates the distance between the take-off position and the destination, i.e.,

(37) $$\begin{array}{l} d_{d}=\sqrt{{\left(x_{0}-x_{d}\right)}^{2}+{\left(y_{0}-y_{d}\right)}^{2}+{\left(z_{0}-z_{d}\right)}^{2}} \end{array}$$

**Figure 4:**
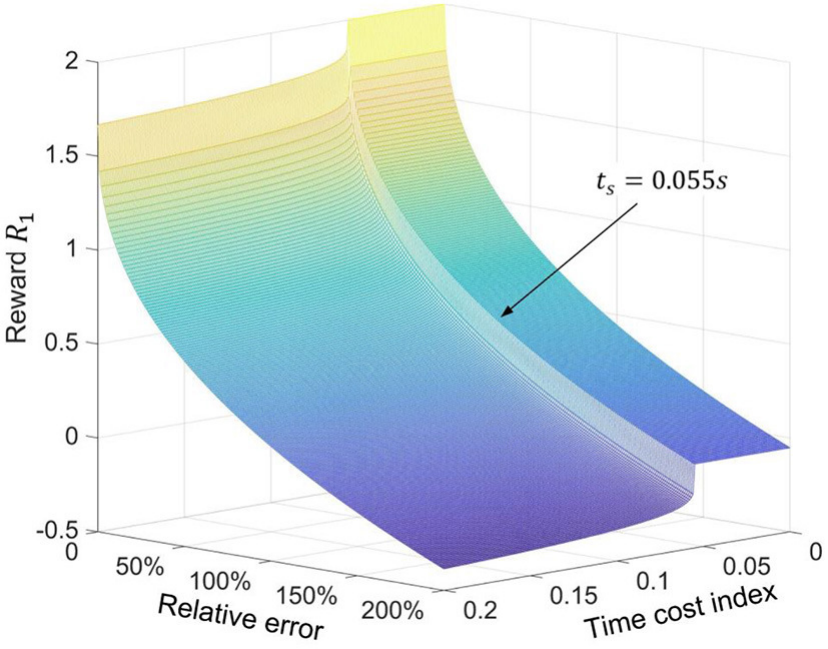
Distribution of the value of the reward function *R*_1_ on the parameter *t*_*s*_ and parameter δ.

Then, the first sub-reward function is designed as follows,

(38) $$\begin{array}{l} r_{1,1}=1-\delta^{0.4} \end{array}$$

where constant-coefficient 0.4 is an empirical parameter by error and trial. For the sub-reward function *r*_1,1_, the smaller the relative error, the faster the reward increases. This character will benefit the convergence of the networks in the proposed algorithm. For the second control target, i.e., reducing the time of football flight, the unit time cost index *t*_*s*_ is defined as follows.

(39) $$\begin{array}{l} t_{s}=t_{f}/d_{m} \end{array}$$

where *t*_*f*_ is the football's flight time and parameter *d*_*m*_ can be found in Equation (34). Then, the second sub-reward function is defined as follows:

(40) $$\begin{array}{l} r_{1,2}=1-{\left(\max\left(t_{s}-t_{0},0\right)\right)}^{0.15} \end{array}$$

where symbol *t*_0_ = 0.055 *s*/*m* is the empirical value based on simulations, which indicates the expected unit time cost. As defined by the sub-reward function *r*_1,2_, the lower the unit time cost index, the higher the value of the reward. Then, the united reward function will be shaped as Equation (41).

(41) $$\begin{array}{l} R_{1}=\frac{14}{9}r_{1,1}+{\frac{4}{9}r}_{1,2,\;}z_{f}=Z_{LB}\;or\;z_{f}=Z_{LH} \end{array}$$

where the value of the reward function *R*_1_ is restricted from 0 to 2 according to the recommended value of the Henderson et al. (2018). Considering the importance of the first control target and the value limitation of the *R*_1_, $\frac{14}{9}$ and $\frac{4}{9}$ are selected the shaping weights for *r*_1,1_ and *r*_1,2_, respectively. Since the different control targets have different sensitivities in reward value, reasonable shape weights are helpful to find the optimal policy that can satisfy multiple control targets. However, these weights in reward shaping usually originate in practical experience. The pretest results also demonstrate that changing the shaping weights value will decrease the proposed controllers' performance. After shaping, the distribution of the reward function *R*_1_ on relative error δ and unit time cost index *t*_*s*_ is shown in Figure 4.

### Scenario 2: Chipping the football over the human wall

**Figure 5:**
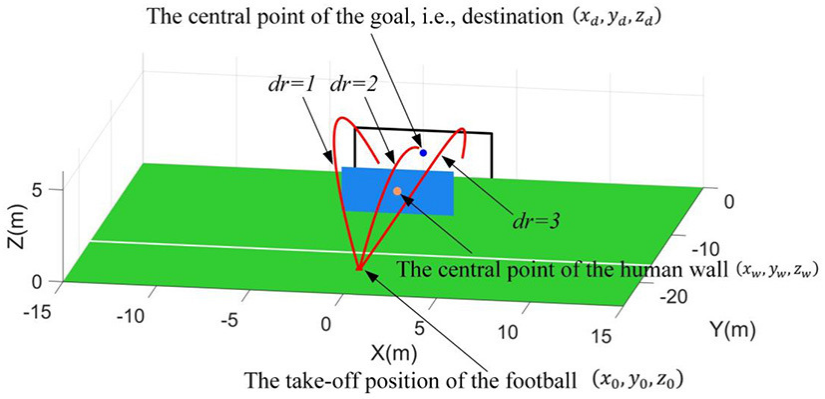
Chipping the football over the human wall.

The schematic diagram of chipping the football over the human wall is shown in Figure 5. In this scenario, the football is required to fly over (rather than through) the human wall and reach at the goal. Indeed, this scenario simulates the free kick situation in the football game. Similar as the first scenario, the action outputted by the RL controller is the football's initial velocities, which are defined in Equation (31). Here, two control targets, i.e., chipping the football into the goal and reducing the time of the football flight, are set for the RL controller.

In this scenario, the goal is defined as perpendicular to the positive Y-axis and the projection of the goal's central point on the X-Y plane is set at the coordinate origin (0, 0, 0). Thus, the central point of the goal will be always regarded as the destination, i.e., (*x*_*d*_, *y*_*d*_, *z*_*d*_) = (0, 0, 1.22) (The height of the goal is 2.44 m based on the FIFA standards). Then, a 2.4 × 6 m human wall parallel to the goal is placed between the goal and take-off position of football. Here, the projection points of the human wall's central point, the goal's central point, and the football's take-off position on the X-Y plane are assumed to be collinear. Thus, the conditions when the football takes off in this scenario, i.e., state *S*_2_, can be described as follows.

(42) $$\begin{array}{l} S_{2}=\left(x_{0},y_{0},z_{0},dr,x_{w},y_{w},z_{w}\right) \end{array}$$

where, the (*x*_0_, *y*_0_, *z*_0_) can be found in Equation (32). The (*x*_*w*_, *y*_*w*_, *z*_*w*_) represents the central point of the human wall. The parameter *dr* represents the specified direction requirement for flight trajectories. Namely, *dr* = 1 is left side of the human wall, *dr* = 2 is top of the human wall, and *dr* = 3 is right side of the human wall. Then, the constraints for the state *S*_2_ are defined as follows. The constraints for take-off position are set as *x*_0_ ∈ [−20, 20] and *y*_0_ ∈ [−15, −25]. Note that *z*_0_ ≡ 0. Based on the free-kick rules, the constraint for the human wall's position is defined as follows,

(43) $$\begin{array}{l} \sqrt{{{(x}_{0}-x_{w})}^{2}+{{(y}_{0}-y_{w})}^{2}}\geq 9.15 \end{array}$$

Due to the human wall, the flight trajectories of footballs are required to specified shapes. Meanwhile, multiple specified direction requirements are considered, which means more functional requirements. Thus, the complexity of this scenario is significantly increased more than the first scenario.

In this scenario, another two termination events should be defined, besides two termination events *z*_*f*_ = *Z*_*LB*_ or *z*_*f*_ = *Z*_*LH*_ described in Section Scenario 1: Passing the football to a moving player. Here, the third termination event triggered by the human wall (*y*_*f*_ = *y*_*w*_) is required. That is, the football bumps into the human wall. Based on the parameter *dr*, the third termination event has three triggering conditions, which can be expressed as follows.

(44) $$\begin{array}{l} \{\begin{array}{l} x_{f}\geq x_{w}-3\;,\text{when dr}=1\text{ and }{\text{y}}_{\text{f}}={\text{y}}_{\text{w}}\;\\ |x_{f}-x_{w}|>3\;or\;z_{f}\leq 2z_{w},\text{when dr}=2\text{ and }{\text{y}}_{\text{f}}={\text{y}}_{\text{w}}\;\\ x_{f}\leq x_{w}+3,\text{ when dr}=3\text{ and }{\text{y}}_{\text{f}}={\text{y}}_{\text{w}} \end{array} \end{array}$$

Then, the fourth termination event indicates that the football reaches at the two-dimensional surface corresponding to the goal, which is written as *y*_*f*_ = 0.

**Figure 6:**
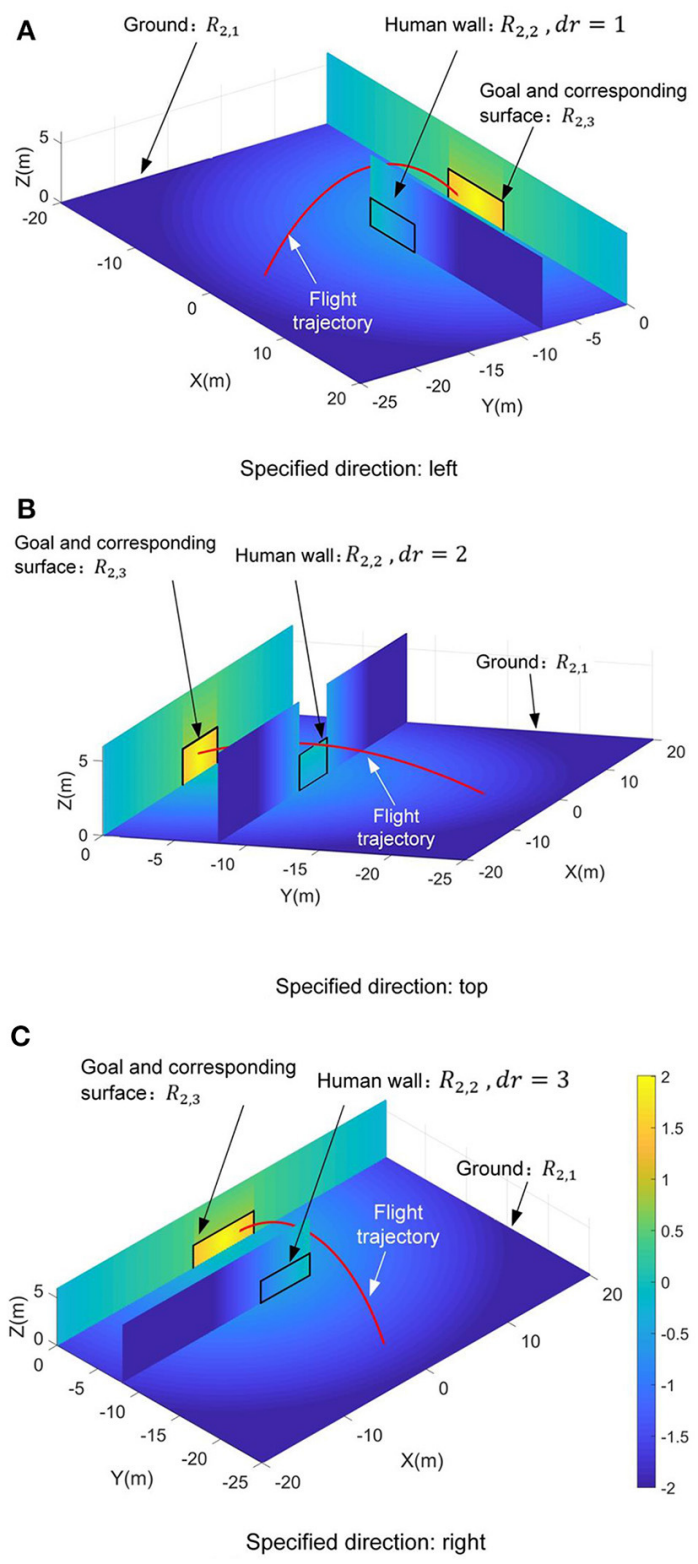
Distributions of the value of the reward function R_2_ on the sports field. **(A)** The specified direction *dr* = 1. **(B)** The specified direction *dr* = 2. **(C)** The specified direction *dr* = 3.

Since the complexity of the control requirements in the second scenario, three independent reward functions, i.e., *R*_2,1_, *R*_2,2_, and *R*_2,3_, are designed respectively depending on the triggering four termination events. Note that triggering the fourth termination event *y*_*f*_ = 0 is the essential precondition for chipping the football into the goal. Thus, only when the fourth termination event is triggered, reducing the time of football flight should be considered, and the relevant reward function *R*_2,3_ is set from 0 to 2. And other reward functions *R*_2,1_ and *R*_2,2_ are defined between −2 and 0 to ensure the coherence of the reward's guidance (see Figure 6). Since each reward function only works on a specified termination event, a simple linear function (i.e., *y* = *kx* + *b*) is also selected as the reward's basic function besides the power function.

When the first and second termination events are triggered, i.e., *z*_*f*_ = *Z*_*LB*_ or *z*_*f*_ = *Z*_*LH*_, the first reward function is designed as follows to guide the football close to the destination.

(45) $$\begin{array}{l} R_{2,1}=-2\delta ,\;z_{f}=Z_{LB}\;or\;z_{f}=Z_{LH} \end{array}$$

where δ can be found in Equation (35). When the third termination event takes effect, that is, the football hits the human wall, the second reward function should guide the ball to fly over the human wall. Based on the definition of the third termination event's triggering conditions in Equation (44), the second reward function can be expressed as follows.

(46) $$\begin{array}{l} r_{2,2}=\{\begin{array}{l} \begin{array}{l} -0.17\left(x_{f}-x_{w}+3\right),\;\\ -0.17\left(|x_{w}-x_{f}|-3\right)-0.5, \end{array}\\ \begin{array}{l} -0.21\left(2z_{w}-z_{f}\right),\;\\ -0.17\left(x_{w}+3-x_{f}\right), \end{array} \end{array}\;\begin{array}{l} \begin{array}{l} dr=1,\;y_{f}=y_{w},x_{f}\geq x_{w}-3\;\\ \;dr=2,\;y_{f}=y_{w},|x_{f}-x_{w}|>3 \end{array}\\ \begin{array}{l} dr=2,\;y_{f}=y_{w},z_{f}\leq 2z_{w}\;\\ dr=3,\;y_{f}=y_{w},x_{f}\leq x_{w}+3 \end{array} \end{array} \end{array}$$

When the fourth termination event is triggered *y*_*f*_ = 0, two independent sub-reward functions are designed for chipping the football into the goal and reducing the time of the football flight, respectively. The first sub-function *r*_2,3*a*_ is used to guide the football toward the goal, which is designed as follows.

(47) $$\begin{array}{l} r_{2,3a}=\{\begin{array}{l} -0.068|x_{f}-x_{d}|+0.75,\;\\ -0.14\left(z_{f}-z_{d}\right)+1.1708,\\ -0.26d+3,\; \end{array}\;\begin{array}{l} y_{f}=0,\;|x_{f}-x_{d}|>3.66\;\\ y_{f}=0,\;z_{f}-z_{d}>1.22\;\\ \;y_{f}=0,\;else\; \end{array} \end{array}$$

here *d* can be found in Equation (36). The second sub-function *r*_2,3*b*_ is used to optimize the flight time. Referring to the Equation (40), it can be expressed as follows.

(48) $$\begin{array}{l} r_{2,3b}=1-{\left(\max\left(t_{s}-t_{0},0\right)\right)}^{0.15},y_{f}=0 \end{array}$$

where the unit time cost index *t*_*s*_ is defined as *t*_*s*_ = *t*_*f*_/*d*_*d*_. The *d*_*d*_ can be found in Equation (37). And *t*_0_ can be found in Equation (40). Then, the third reward function are shaped as Equation (49).

(49) $$\begin{array}{l} R_{2,3}=\frac{1}{2}r_{2,3a}+\frac{1}{2}r_{2,3b}\;,\;y_{f}=0 \end{array}$$

where $\frac{1}{2}$ and $\frac{1}{2}$ are the shaping weights. Note that the value of the third reward function is designed to be larger than the first and second. This design can effectively guide the football reaching to the goal. Under the requirements of three specified directions, the distributions of the value of the reward function *R*_2_ on the sports field are shown in Figure 6. Actually, reward function design is an experienced-based work (Dewey, 2014; Henderson et al., 2018; Silver et al., 2021). The constant-coefficients of the Equation (38), Equation (40), Equation (41), and Equations (45-49) are all determined by error and trial. And the pretest results verify that the proposed reward functions have strong guidance for optimizing control strategy under the effects of these constant-coefficients.

## Comparison and discussion

In this section, the advantages of the immediate-return RL algorithm for atypical MDPs will be discussed and demonstrated. Meanwhile, PPO (the representative of the stochastic policy algorithms) and DDPG (the representative of the deterministic policy algorithms) are chosen as the references for the proposed algorithm. All these algorithms will train corresponding controllers for two football flight scenarios. Then, the advantages of the proposed algorithm will be discussed and analyzed from the training process, training results (i.e., the performance of the controllers), and computing resource usage by comparing with these reference algorithms.

### Training process

**Figure 7:**
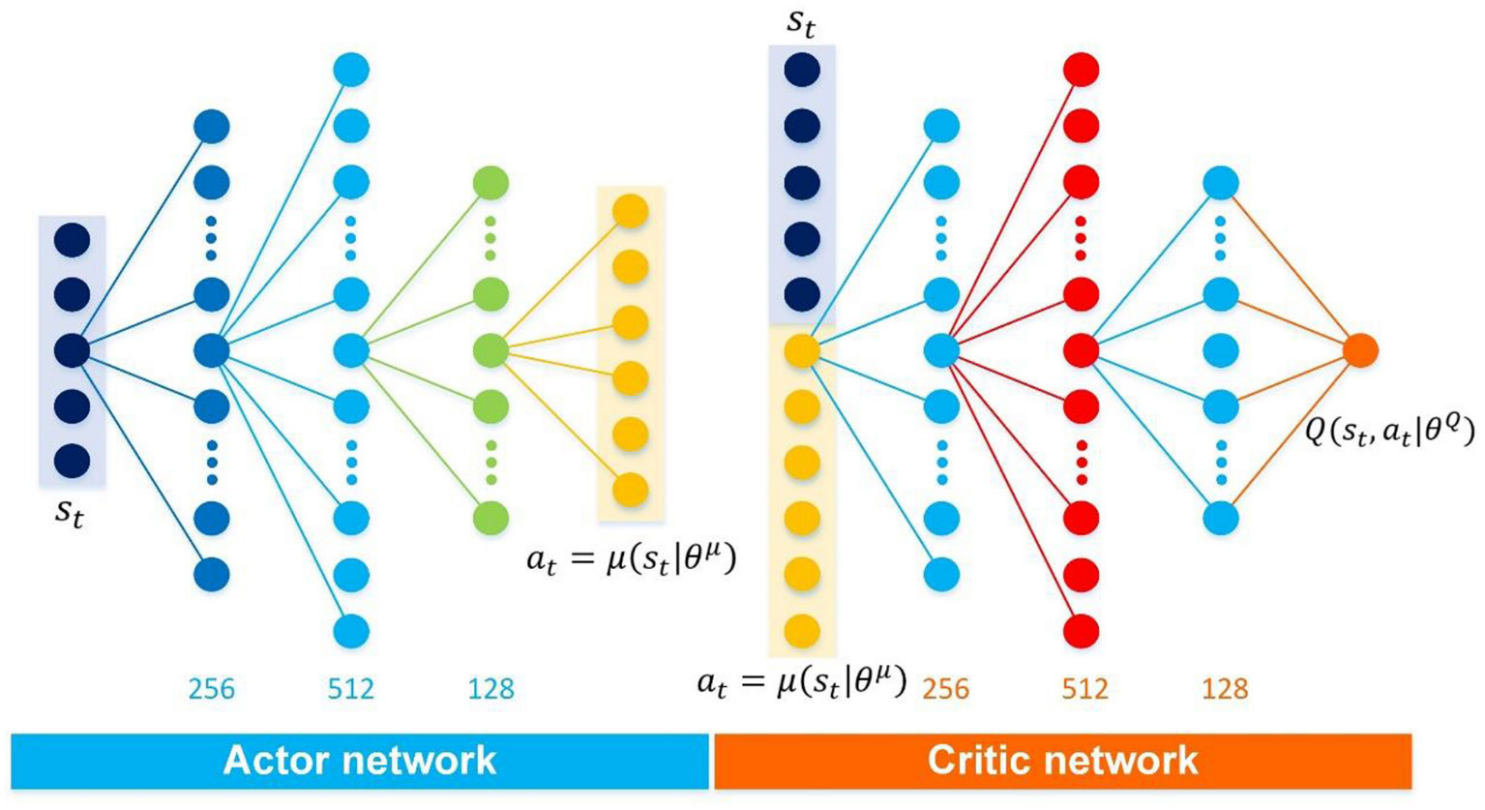
Detailed network architectures of the proposed algorithm.

For the control problems of the football trajectory, the proposed algorithm's detailed network framework is designed in Figure 7, including an independent actor network and an independent critic network. Here, the proposed algorithm's actor network and critic network have the same hidden layers and node numbers, i.e., the same network architectures. Indeed, each independent network in the three discussed algorithms shares the same network architectures to avoid the influence of the network architectures on the test results. Similarly, all discussed algorithms use the same reward function designed in Section Illustration examples: Football trajectory control for different scenarios. Furthermore, it should be noted that different deep RL algorithms have different sensitivities to hyperparameters (Henderson et al., 2018). Based on the trial and error and the experience of Dewey (2014), Henderson et al. (2018); and Silver et al. (2021), the detailed hyperparameters of each algorithm are selected (see Table 4). Under the premise of ensuring the algorithm's performance, each algorithm's hyperparameters are set to the same value.

**Table 4:** The hyperparameters of the discussed deep RL algorithms.

	**Learning rate for actor**	**Learning rate for critic**	**Discount factor**	**Soft target updates**
The proposed algorithm	1e-4	1e-4	\	\
DDPG	1e-4	1e-4	0.9	0.01
PPO	5e-6	1e-5	0.9	\

**Figure 8:**
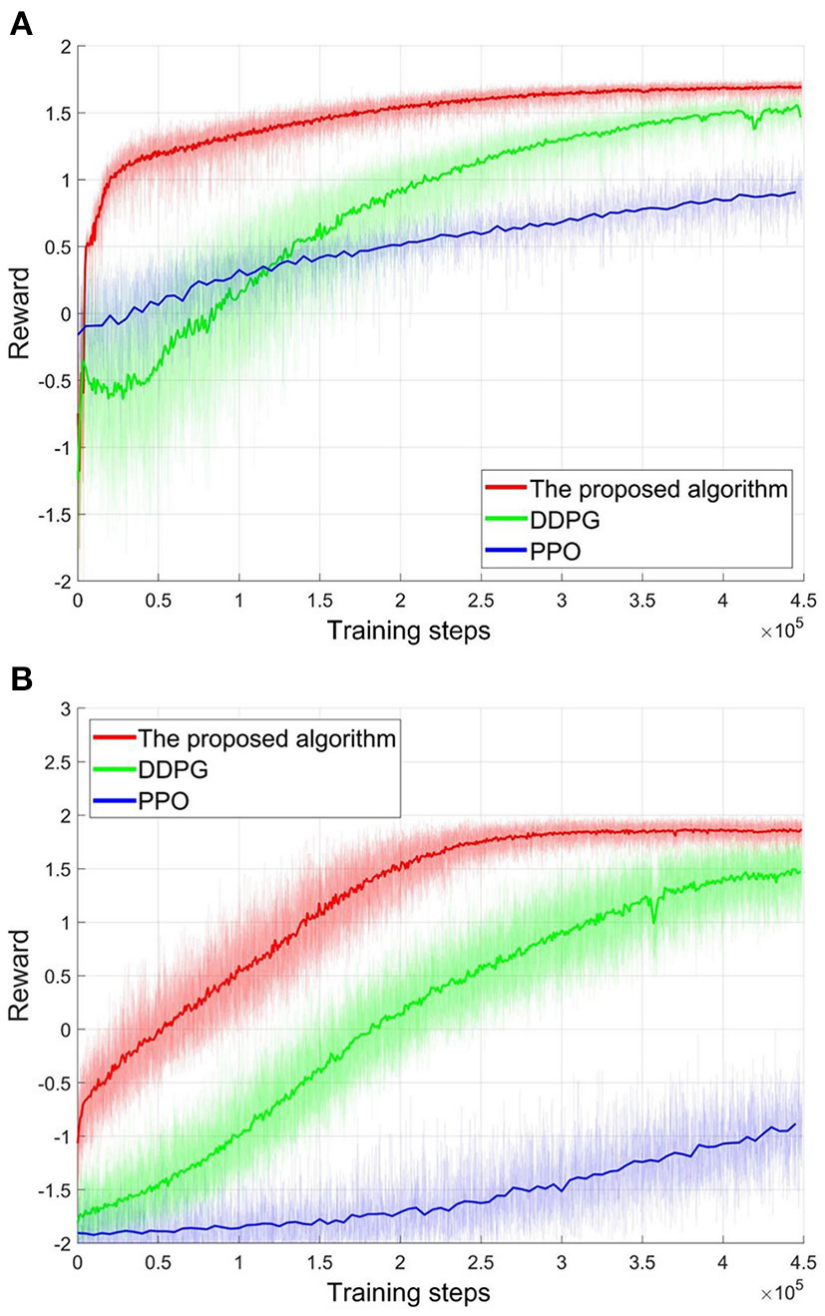
The reward curves of different algorithms. **(A)** The first scenario. **(B)** The second scenario.

Then, all algorithms, i.e., the proposed algorithm, DDPG, and PPO, will train the corresponding controllers for these two scenarios. Here, the learning efficiency of the algorithm can be evaluated by the consumption of the training steps. After 450,000 training steps, all reward curves in these two scenarios are shown in Figure 8. In both scenarios, the reward curves of the proposed algorithm (red line in Figure 8) converge to the high-level reward value after 300,000 training steps. Then, the suitable controllers can be obtained. Although the reward value of the DDPG algorithm also has risen during training (green line in Figure 8), DDPG's learning efficiency is worse than the proposed algorithm from the perspective of convergence speed. As shown in Figure 8, DDPG needs about 450,000 training steps to converge the reward curves. That is, the learning efficiency of the proposed algorithm is 1.5 times that of the DDPG. And the convergency reward value of the DDPG is also less than the proposed algorithm. As a stochastic policy algorithm, PPO shows poor learning ability in football trajectory control. As show in Figure 8, 450,000 training steps do not allow the PPO to converge. Actually, PPO can also be converged after consuming about 1,500,000 training steps. That is, the learning efficiency of the proposed algorithm is 5 times that of the PPO. Furthermore, the final convergency reward values of the PPO are far less than the proposed algorithm. Note that the more training steps, the more samples are required. Thus, the training process confirms the analysis in Section Atypical MDPs: Definition and characteristic analyses. That is, PPO's learning efficiency is low in the atypical MDPs, as estimating a state-value requires more samples. The above training process demonstrates that the proposed algorithm converges faster and consumes fewer samples compared to DDPG and PPO. That is, the proposed algorithm shows better learning efficiency. Actually, it is a significant advantage for the proposed algorithm, as the samples are difficult to obtain in many atypical MDP cases.

### Controller's performance

In this section, the performance of the controllers will be analyzed from three aspects, i.e., accuracy, unit time cost, and reliability. As described in Section Illustration examples: Football trajectory control for different scenarios, two control targets, i.e., shooting the football to the destination and reducing the time of football flight, are considered for each scenario. Thus, accuracy and unit time cost are the core index for evaluating the control performance of the controllers. Actually, the control performance is closely related to the value function's estimation bias. Besides, the considered aerodynamic model of football is an uncertain environment. That is, the football trajectory may be completely different under the same state-action pair, bringing a high variance for the value function. To evaluate the effect of variance caused by the uncertain environment on the controller, the reliability is set as another index for the controller's performance.

Here, Monte Carlo tests are applied to analyze the control performance of the controllers. In each scenario, 1,000 independent state will be chosen randomly and a set of initial velocities will then be generated by the tested controller for each chosen state. Then, only one flight trajectory will be generated for the chosen state and the outputted initial velocities. Here, the effective rate of control *Re* is defined as follows to evaluate the accuracy of the RL controller.

(50) $$\begin{array}{l} Re=N_{Re}/1000 \end{array}$$

where *N*_*Re*_ is the number of the flight trajectories successfully controlled in 1000 tests.

For the first passing scenario, if the relative error δ is less than 5%, the flight control will be regarded as success. Here, the relative error δ is defined as follows.

(51) $$\begin{array}{l} \delta =\frac{\sqrt{{{(x}_{d}-x_{f})}^{2}+{{(y}_{d}-y_{f})}^{2}}}{\sqrt{{{(x}_{d}-x_{0})}^{2}+{{(y}_{d}-y_{0})}^{2}}} \end{array}$$

**Figure 9:**
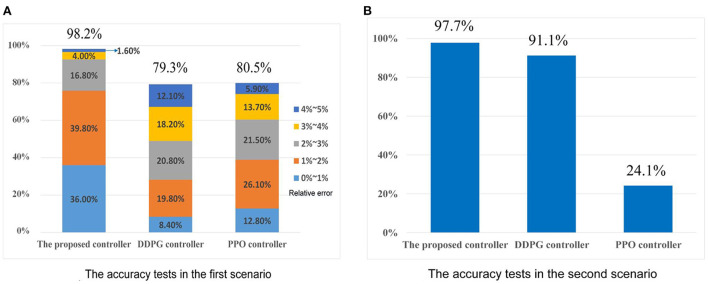
The accuracy tests. **(A)** The accuracy test's results in the first scenario. **(B)** The accuracy test's results in the second scenario.

As shown in Figure 9A, the effective rate of control *Re* of the proposed controller in the first scenario, i.e., passing the football to a moving player, is 98.2%. In particular, there are 36.0% tests with relative error less than 1%, 56.6% tests with relative error from 1 to 3%, and 5.6% tests with relative error between 3 and 5%. Under the same tests, the DDPG controller's *Re* is 79.3%, and the PPO controller's *Re* is 80.5%. For the second scenario, scoring goals are regarded as the successful controls. The effective rate of control *Re* of the proposed controller for chipping the football over the human wall is 97.7% (see Figure 9B). Meanwhile, the DDPG controller's *Re* and PPO controller's *Re* are 91.1 and 24.1%, respectively. Compared to DDPG and PPO, the good accuracy of the proposed controller is verified in both two scenarios.

**Figure 10:**
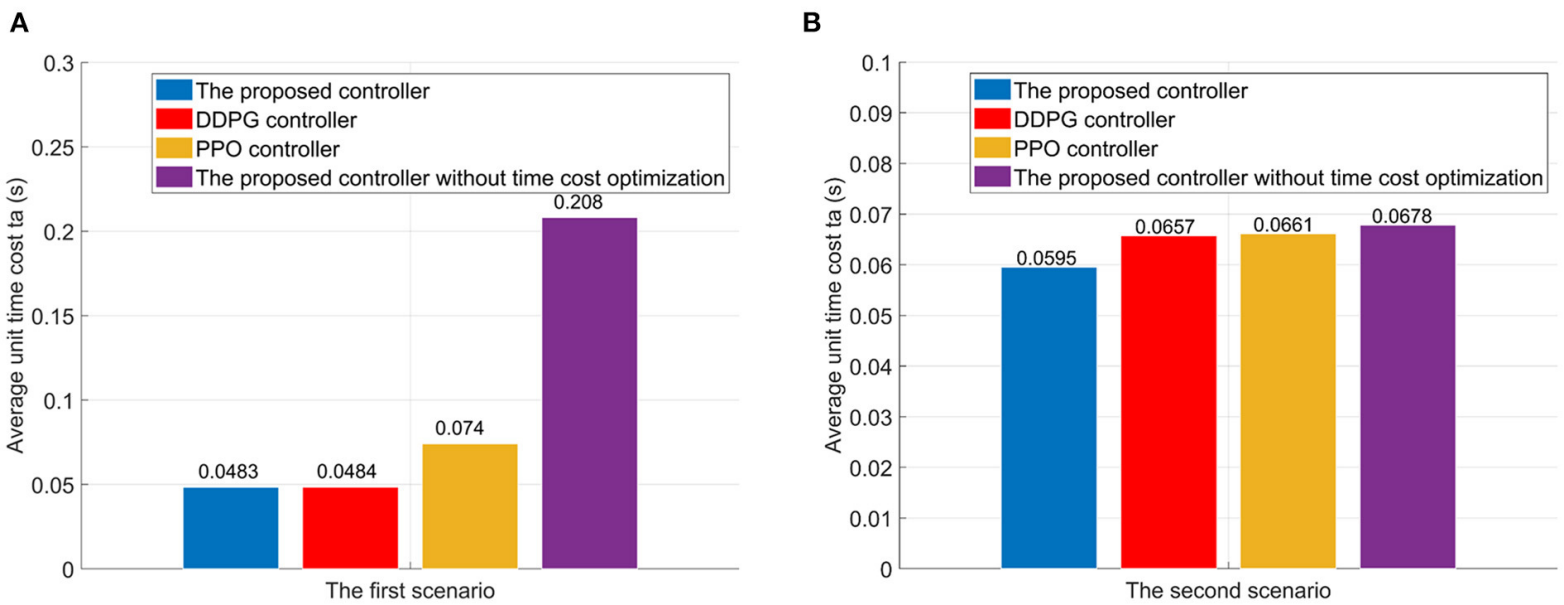
The average unit time cost in flights. **(A)** The first scenario. **(B)** The second scenario.

Based on 1,000 Monte Carlo tests, the average unit time cost *t*_*a*_ of 1,000 tests is used to evaluate the unit time cost, which can be written as.

(52) $$\begin{array}{l} t_{a}=\sum_{1}^{1000}t_{s}/1000 \end{array}$$

here, *t*_*s*_ is the unit time cost index, which can be found in Equation (39). For the sake of comparison and evaluation, the proposed controllers without the time cost optimization are also trained for two scenarios. In the first scenario, the proposed controller reduces the average unit time cost *t*_*a*_ from 0.2080s to 0.0483s, comparing to the proposed controller without the time cost optimization (see Figure 10). Meanwhile, the DDPG controller can reduces the unit time cost *t*_*a*_ to 0.0484s. And the PPO controller can reduce the unit time cost *t*_*a*_ to 0.074. In the second scenario, adding the time optimization has little effect on flight time. However, the unit time cost of the proposed controller is the lowest compared to the DDPG and PPO controllers.

As analyzed in Section Limitations of existing RL algorithms in the atypical MDPs, the estimated value functions in existing RL algorithm, e.g., DDPG and PPO, is biased due to the TD learning method. Meanwhile, the sampling error *err*(*s*_*t*_) can further increase the estimation bias of the state-value function for the stochastic policy algorithms, as analyzed in Section Atypical MDPs: Definition and characteristic analyses. These estimation biases have adverse effects on the policy update. However, due to the average reward method (see Section The immediate-return RL algorithm), an unbiased target *Q*-value is provided for the proposed algorithm. Thus, the disadvantages of the estimation bias can be overcome. According to the above test data, the effective rate of control *Re* of the proposed controller in the first scenario is increased by 18.9% than the DDPG controller and increased by 17.7% than the PPO controller. In the second scenario, the effective rate of control *Re* of the proposed controller is increased by 6.6% than the DDPG controller and increased by 73.6% than the PPO controller. The proposed algorithm also shows better time cost optimization than DDPG and PPO in both two scenarios. Thus, the high accuracy and low unit time cost of the proposed controllers can be verified. This also means that the immediate-return RL algorithm has better performance than existing RL algorithms in deal with the atypical MDPs.

**Figure 11:**
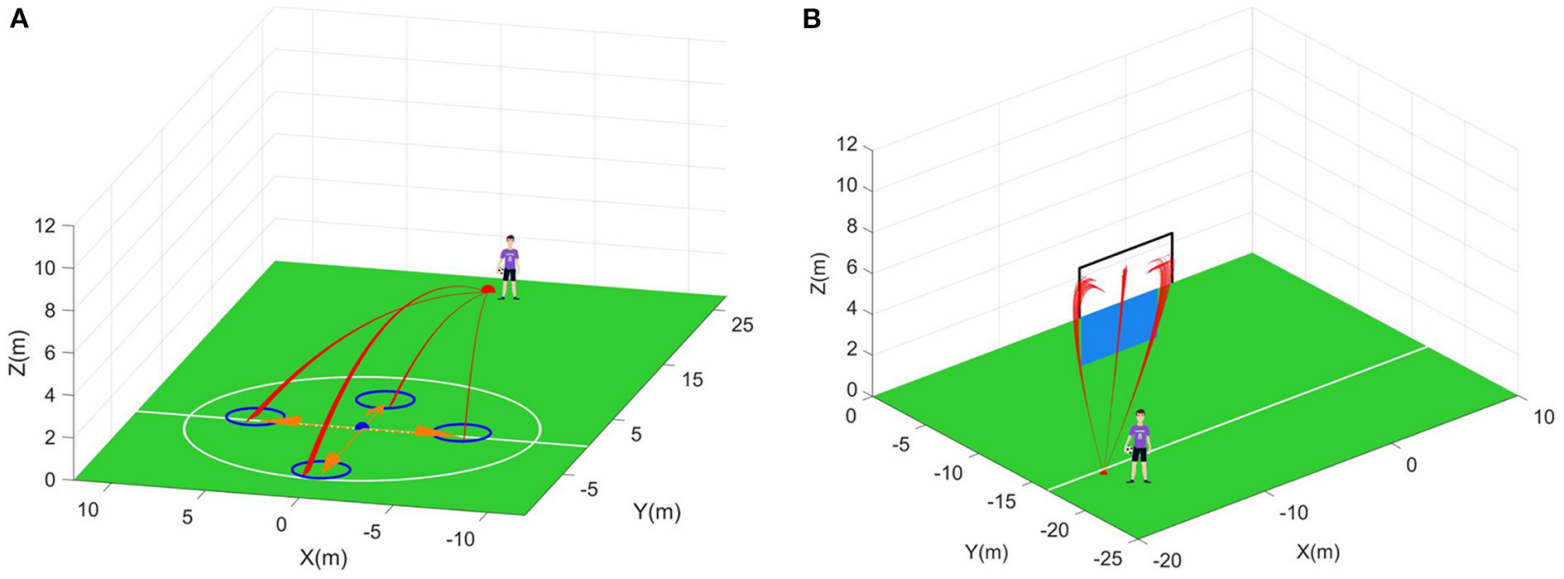
Reliability tests. **(A)** The first scenario. Blue circle is the allowed landing range. **(B)** The second scenario. Blue plane is the human wall. Black wireframe is the goal.

In the reliability tests, several specified states will be chosen for the tested controllers in each scenario (see Figure 11). For each chosen state, the only set of definite initial velocities will be outputted by the corresponding controller. Then, in the uncertain environment, 200 different flight trajectories will be generated based on the same chosen states and the same initial velocities. To evaluate the reliability of the controllers, the reliable rate *Rr* is defined as the effective rate of control of the repeated 200 tests on the same chosen state, which is written as Equation (53)

(53) $$\begin{array}{l} Rr=N_{Rr}/200 \end{array}$$

where *N*_*Rr*_ is the number of the flight trajectories controlled successfully in 200 reliability tests.

**Figure 12:**
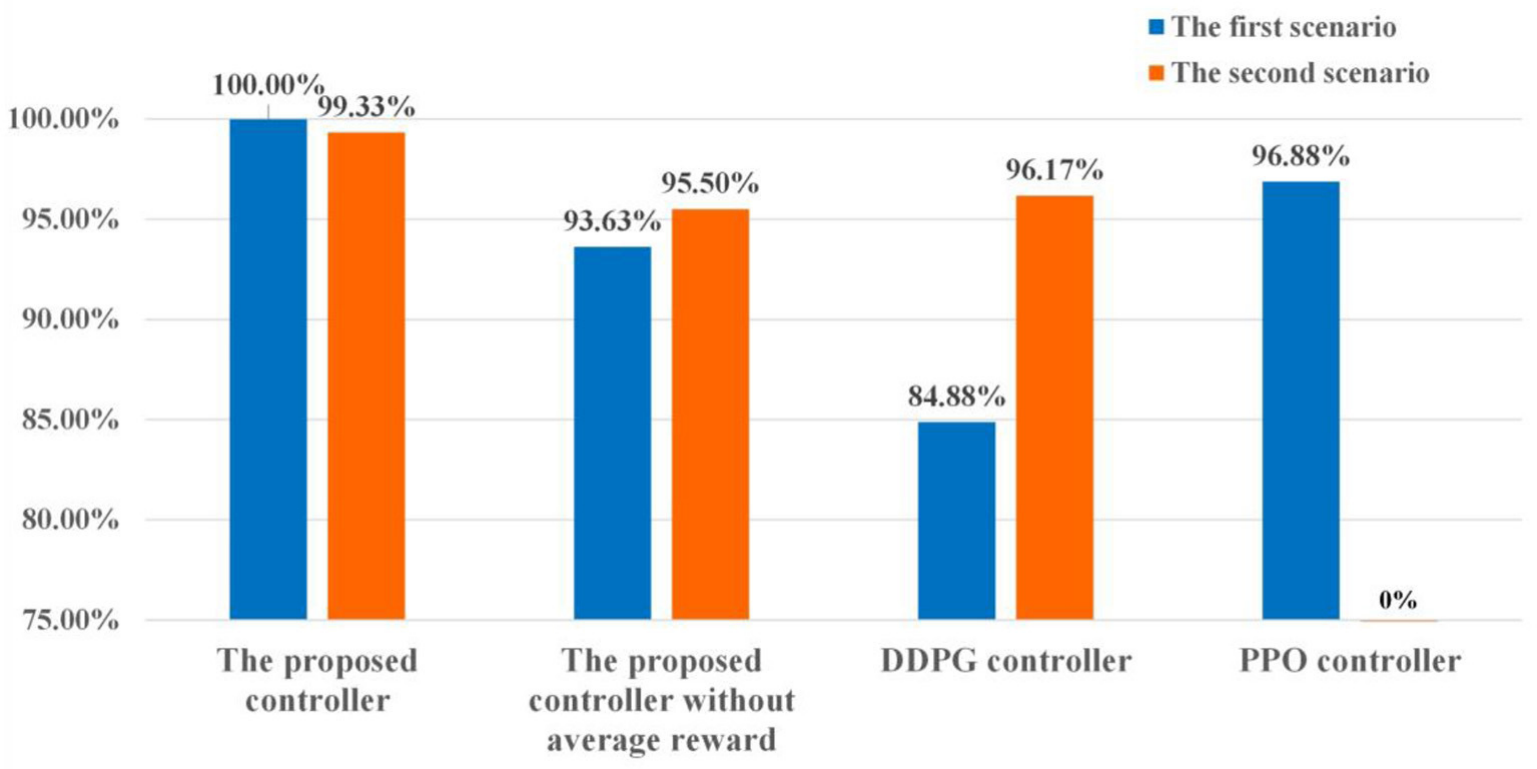
The results of the reliability tests.

In the first scenario, a point is selected as the initial position of the moving player. The moving player is assumed to move along the four directions marked by the orange arrows in Figure 11A now. That is, four states are chosen for the tested controllers. According to Figure 12, the average reliable rate of the proposed controller for the first scenario is 100.00%. The average reliable rates of the DDPG controller and PPO controller are 84.88 and 96.88% respectively. In the second scenario, one point is selected as the initial take-off position of the football (Figure 11B). In this initial take-off position, three specified directions where the football flies over the human wall are tested. That is, three states are constructed in the second scenario to test controllers. In this scenario, only 4 trajectories are not control in the total of 600 trajectories under the effect of the proposed controller. The average reliable rate of the proposed controller is 99.33%. The DDPG's average reliable rate in the second scenario is 96.17%. Notice that the PPO controller do not finish the reliability tests due to its terrible control policy.

The reliability in uncertain environments is also an important index to evaluate the controller's performance. In this paper, the aerodynamic model of football with parameter uncertainties is regarded as the uncertain environment. Due to the strong non-linear of the football model, there may be more than one set of initial velocities to meet the requirements of the specified flight purpose. Meanwhile, the same initial velocities may generate different trajectories due to the parameter uncertainties. Thus, high reliability means that the expected reward under the specified state-action pair can be estimated accurately. And the controller can find a good set of initial velocities from multiple possible initial velocities, reducing the effects of the parameter uncertainties on the flight trajectories. According to test data in Figure 12, the reliabilities of the proposed controllers are approaching or equal to 100% in both two football flight scenarios, which is significantly better than DDPG and PPO controllers. The above results verify that the proposed controllers have great reliability and can find the best initial velocities to resist the adverse effects of uncertain environments. As analyzed in Section The immediate-return RL algorithm, the great reliability of the proposed controllers come from the average operation for reward. For the sake of comparison, two controllers based on the proposed algorithm without using the average reward are also trained. As shown in Figure 12, the reliable rate of the controller without the average reward is reduced by 6.37% in the first scenario and reduced by 3.83% in the second scenario. Numerical results indicate that the average reward method can improve the reliability of the controller.

### Computing resource usage

As analyzed in Section Complexity analysis, compared to existing RL algorithms, the network framework of the immediate-return RL algorithm is greatly simplified, and its complexity is reduced significantly. That is, when solving the same problem in the atypical MDPs, the immediate-return RL algorithm may consume fewer computing resources than existing RL algorithms. Therefore, taking the first scenario of the football trajectory control as an example, the computational resource requirements of different algorithms, i.e., immediate-return RL algorithm, DDPG, and PPO, are analyzed.

In these tests, the hardware is a normal computer with Intel I5 8600k processor and Nvidia GPU RTX2060. And all networks are built by the Tensroflow. For unity, 300,000 training steps are provided for each tested algorithm. Then, the computing resources consumed by three tested algorithms are shown in Table 5. As can be seen, the immediate-return RL algorithm reduces the CPU utilization by 18.8%, the memory utilization by 26.3%, computing time by 29.4%, and size of the networks by 25.0% than the DDPG. Compared to PPO, the immediate-return RL algorithm also reduces the CPU utilization by 13.3%, the memory utilization by 12.5%, computing time by 2.0%, and size of the networks by 14.2%. It should be noticed that the number of training steps is limited to 300,000 in all tests. However, the computing resource usage of the algorithms also depends on the number of training steps required. Since the convergence speed of both DDPG and PPO is slower than the proposed algorithm, they require much more training steps than the proposed algorithm in actuality (see Figure 8). As analyzed in Section Training process, the number of training steps used by the proposed algorithm is 66.7% of the DDPG and 20% of the PPO. That is, the advantage of proposed algorithm in computing time is greater than that shown in the Table 5. Thus, the test data demonstrates that, when dealing with the same problem in the atypical MDPs, the immediate-return RL algorithm trains faster, occupies less CPU and Memory, and generates fewer networks than existing RL algorithms. Furthermore, it should be noted that the transfer processes of data between CPU and GPU also consumes computing resources. The simulations of the football flight also affect the usage of computing resources. Thus, the differences between the comparison results and the theoretical analysis in Section Complexity analysis are acceptable.

**Table 5:** Computing resources usage tests.

	**CPU utilization**	**Memory utilization (GB)**	**Computing time (s)**	**Size of the networks weights (KB)**
The proposed algorithm	26%	1.4	2,359	4,682
DDPG	32%	1.9	3,342	6,243
PPO	30%	1.6	2,408	5,455

## Conclusion

The atypical MDPs exist widely in the engineering field, which involves one state transition with continuous action space. The control goal of the atypical MDPs is to maximize the immediate returns. However, the existing RL algorithms are designed for standard MDPs to maximize long-term returns. Thus, they can cause significant estimation errors for the value function and a waste of computing resources when dealing with the atypical MDPs. To solve such problems, this paper analyzes the characteristics of the atypical MDPs systematically and explains the differences between estimating the state-value function and estimating the action-value function. On this basis, the immediate-return RL algorithm was proposed to deal with the atypical MDPs. In the proposed algorithm, the method of average reward is developed to provide the unbiased and low variance target Q-value. Thus, the problems of large estimation errors can be overcome. And a newly designed network framework is designed for the proposed algorithm, which can significantly reduce computing resource usage. Then, two scenarios of the football trajectory control, i.e., passing the football to a moving player, and chipping the football over the human wall, are designed as the benchmark to test the algorithms designed for the atypical MDPs. Numerical results demonstrate that the learning efficiency of the proposed algorithm is 1.5 times that of the DDPG and 5 times that of the PPO. For the controllers based on the proposed algorithm, their effective rates of control are more than 97.7%, and their reliabilities are approaching 100%. Such performance is far superior to DDPG and PPO. As the proposed controller increases the shot's accuracy significantly, it can promote the development of high-level football robots in the Robot world cup. Furthermore, the proposed algorithm can also consume fewer computing resources than existing RL algorithms. Thus, the immediate-return RL algorithm has higher learning efficiency, higher performance, and lower computing resource usage than the existing RL algorithms, such as PPO and DDPG.

It should be pointed out that the immediate-return RL algorithm can output only one determined action. This determined value can be seen as the best solution according to the specified rewards function. However, a single best solution based on the specified rewards function is impractical for many complex engineering problems (e.g., strongly non-linear dynamic system with parameter uncertainties). As one focus of the future work, efforts will be made to improve the algorithm to find a proper basin which corresponds to the specified scenario. After that, the action output shall be more practical. In the future, we will devote ourselves to expand the use of the proposed immediate-return RL algorithm and achieve more engineering applications, such as stamping process, directional blasting, approximations of the compound Poincaré maps, etc.

## Data availability statement

The original contributions presented in the study are included in the article/supplementary material, further inquiries can be directed to the corresponding authors.

## Author contributions

ZP contributed to algorithm design and development, data analysis, and writing the first manuscript. GW contributed to the study conception and design and supervised the overall study. ZT assisted in implementing the code and collecting datasets. SY guided the research and provided a critical review. XH collected related literature and assisted in the data processing. All authors reviewed and approved the final manuscript.
